# Bland–Altman Plot for Censored Variables

**DOI:** 10.1002/sim.70147

**Published:** 2025-06-05

**Authors:** Anne Lotz, Thomas Behrens, Karl‐Heinz Jöckel, Dirk Taeger

**Affiliations:** ^1^ Institute for Prevention and Occupational Medicine of the German Social Accident Insurance (IPA), Institute of the Ruhr University Bochum Germany; ^2^ Institut für Medizinische Informatik, Biometrie und Epidemiologie, Universitätsklinikum Essen, Universität Duisburg‐Essen Germany

**Keywords:** difference plot, limited variable, nondetects

## Abstract

The comparison of two measurement methods turns out to be a statistical challenge if some of the observations are below the limit of quantification or detection. Here we show how the Bland–Altman plot can be modified for censored variables. The reference lines (bias and limits of agreement) in the Bland–Altman plot have to be estimated for censored variables. In a simulation study, we compared three different estimation methods: Restricting the data set to fully quantifiable pairs of observations (complete case analysis), naïvely substituting missing values with half of the limit of quantification, and a multiple imputation procedure based on a maximum likelihood approach for bivariate lognormally distributed variables with censoring. The results show that simple ad‐hoc solutions may lead to bias in the results when comparing two measurement methods with censored observations, whereas the presented multiple imputation approach of the Bland–Altman method allows adequate consideration of censored variables. The method works similarly for other distribution assumptions.

## 1 Introduction

The purpose of this article is to describe and evaluate a new approach to draw a Bland–Altman plot in the case of censored variables, for example, due to some values falling below the limit of detection (LOD) of a laboratory method. The Bland–Altman plot is a visual analysis tool to compare two methods that measure a continuous outcome on the same scale [1, 2, 3]. In case of censored variables, the following questions arise: How can the difference and the mean of two observations be appropriately plotted when one or both observations are censored? How can the reference lines, such as the bias line, usually estimated by the mean difference $\bar{d}$, and the 95% limits of agreement, usually estimated using the standard deviation of the mean difference s as $\bar{d}\pm 2s$, be estimated?

If we want to use the notations of the analysis of missing data, we can describe the mechanism causing censored observations that fall below the LOD as not at random. The probability of an observation being censored depends on the underlying value. Unlike missing data, censored observations include information about their values, indicating a range of possible values. It follows that we can improve our estimates of the reference lines of the Bland–Altman plot if we consider the information about the range of the censored observations together with the information given by the observed data.

When analysing censored data, a variety of different methods can be found in the literature. Implementing these methods requires varying levels of effort. The simplest methods are the complete case analysis or naïve imputation procedures. More complex methods include maximum likelihood and multiple imputation procedures. As far as we know, the suitability of these methods for creating Bland–Altman diagrams has never been systematically investigated.

In the presence of missing data, complete case analysis can lead to valid estimators under specific conditions, for example, when the missing data mechanism is completely at random [4]. However, this condition does not apply to censored data, where the missing data mechanism is not at random. It has been demonstrated that estimators derived from the analysis of univariate statistics [5, 6] or regression models with left‐censored independent variables [7, 8] are biased. The situation differs when the censoring impacts only the dependent variables in a regression model, leaving the independent variables uncensored. In such cases, complete case analysis produces unbiased estimators, although with decreased efficiency compared to alternative methods [9, 10].

Naïve imputation methods, such as substituting left‐censored observations with the limit of quantification, half the limit of quantification, or the square root of two times the limit of quantification, are prevalent ad hoc methods due to their simplicity and ease of application. However, in studies comparing the estimation of univariate statistics [5, 6], regression models [7, 8, 11] or correlations [7, 12, 13], the naïve imputation methods are usually inferior to other estimation methods. Helsel [14] indicates that substitution is generally unsuitable for analyzing left‐censored observations. Despite the poor estimation properties, naïve imputation methods remain in use because they are straightforward to implement. Because of the method's practical relevance, we have integrated a variant of it into our comparative analysis.

Maximum likelihood methods, while more computationally complex, generally exhibit superior estimation properties across various statistical models, including univariate statistics [5, 15], regression models [8, 16], generalized linear models [9, 17], and mixed‐effects models [18]. In this study, we will use and expand a maximum likelihood approach that was originally developed for correlation analyses [13]. Assuming a bivariate normal distribution, this method enables us to estimate the distribution parameters and subsequently calculate the values of the reference lines of the Bland–Altman plot, as detailed in the methods section.

Multiple imputation is another recommended technique for handling censored observations. The principal idea behind multiple imputation is to use the distribution of available data to draw a set of likely values for the absent data, factoring in random variations to account for uncertainties [19]. The advantage of multiple imputation methods is that the newly created data sets can be employed in various statistical analyses, provided a suitable multiple imputation method has been found. The estimators obtained for each imputed data set can be combined according to the procedures described by Rubin's rule [19] and usually lead to estimators with recommendable properties. For examples for the evaluation of the estimation properties of multiple imputation methods, refer to [5, 6] for univariate estimation methods [8, 11, 16, 20], for regression models, and [9] for generalized linear models. We are not aware of any multiple imputation approaches for creating Bland–Altman plots. Here, we use multiple imputation methods to obtain a graphical representation of the censored observations in the Bland–Altman plot.

In the following article, we present a multiple imputation approach to construct a Bland–Altman plot for censored observations (Section 2). We compare this method with simple ad‐hoc methods such as complete case analysis and a naïve imputation approach of the censored observations with half the censoring limit in a simulation study (Section 3). We then show how these methods are applied in two examples, one using allergy data and the other using welding fume measurement data. We provide codes in R to draw a Bland–Altman plot for censored observations under the assumption of a bivariate lognormal distribution in the Supporting Information.

## 2 Multiple Imputation Approach of the Bland–Altman Plot for Observations From a Censored Bivariate Normal Distribution

The elements of the Bland–Altman plot can be divided into two components, consisting of (1) the observations, plotted as the difference and arithmetic mean of the pairs of observations, and (2) the reference lines, consisting of the bias line and the limits of agreement. For censored observations, these components cannot be calculated using the formulae given by Bland and Altman [2]. As a basic idea for creating Bland–Altman plots in the case of censoring, we suggest using a multiple imputation approach for censored observations. The multiple imputation approach should use all available information from the uncensored observations, the censoring limits, and any assumptions about the distribution of the pairs of observations to impute the censored values with a set of probable observations. From the multiply imputed values, the difference and arithmetic mean of the censored pairs for the figure can be calculated according to the formulae provided by Bland and Altman [2]. The reference lines of the Bland–Altman plot can be estimated in two ways. In the first approach, the reference lines are calculated according to the Bland–Altman formulae [2] in each imputed data set. The estimates are then summarized according to equations provided by Rubin [19] to obtain final estimates for the reference lines. In the second approach, the reference lines are estimated directly from the estimated distribution of all pairs of observations, and a bootstrap approach [21] is used to account for the uncertainty of the distribution estimate in the estimates of the reference lines. The Bland–Altman plot can then be created using the fully observed and multiply imputed observation pairs and the estimated reference lines.

In the following, we will methodically present this outlined multiple imputation approach for Bland–Altman plots with censoring for two specific scenarios in detail. In both scenarios, it is assumed that all pairs of observations originate from a censored bivariate normal distribution. In the first scenario, the observations are left‐censored with a fixed censoring limit. In the second scenario, the observations are left‐ and interval‐censored with multiple censoring limits. In both scenarios, the parameters of the assumed model are estimated. We will apply a maximum likelihood combined with a bootstrap approach in the following way: In the bootstrap samples, the unknown parameters of the bivariate normal distribution are estimated with a maximum likelihood method. The applied maximum likelihood estimation of a censored bivariate normal distribution with simple‐censoring thresholds was originally developed by Lyles, Williams, and Chuachoowong [13]. In the second scenario, this maximum likelihood estimation method is extended for variables with multiple left‐ and interval‐censoring. The estimates of the parameters of the bivariate normal distribution are used to estimate the reference lines of the Bland–Altman plot. Afterwards, the estimates of the bootstrap samples are combined to final bootstrapped estimates. The bootstrapped estimators of the model are used to multiple impute the censored observations of the sample. Finally, the Bland–Altman plot is drawn with the difference and arithmetic mean of the observed uncensored pairs of observations, the multiple imputed censored pairs of observations, and the estimated reference lines.

### 2.1 Scenario 1: Bland–Altman Plot for Single‐Left Censored Variables

In this scenario, we will describe a method to draw a Bland–Altman plot for paired observations with single‐left censoring. Let X and Y denote two random left‐censored variables, and the thresholds for censoring are $C_{x}$ for X and $C_{y}$ for Y. We observe a random sample $(x_{i},y_{i}),i=1,\ldots ,n$. We assume that $X^{*}$ and $Y^{*}$ are the corresponding latent variables of X and Y with

$$x_{i}=\left\{\begin{array}{ll} x_{i}^{*} & \text{if}\;x_{i}^{*}>C_{x}\\ \text{left‐censored} & \text{if}\;x_{i}^{*}\leq C_{x} \end{array}\right.$$

and

$$y_{i}=\left\{\begin{array}{ll} y_{i}^{*} & \text{if}\;y_{i}^{*}>C_{y}\\ \text{left‐censored} & \text{if}\;y_{i}^{*}\leq C_{y} \end{array}\right.,$$

and that $\left(X^{*},Y^{*}\right)$ follow a bivariate normal distribution with the parameter vector $\theta ={\left(\mu_{x},\mu_{y},\sigma_{x}^{2},\sigma_{y}^{2},\rho \right)}^{T}$.

(1) $$\left(\begin{array}{l} X^{*}\\ Y^{*} \end{array}\right)∼𝒩\left(\left(\begin{array}{l} \mu_{x}\\ \mu_{y} \end{array}\right),\left(\begin{array}{ll} \sigma_{x}^{2} & \rho \sigma_{x}\sigma_{y}\\ \rho \sigma_{x}\sigma_{y} & \sigma_{y}^{2} \end{array}\right)\right)$$

Next, we start the bootstrap method by resampling the sample of paired observations and estimating θ and the reference lines of the Bland–Altman plot as described below in each bootstrap sample.

Lyles, Williams, and Chuachoowong [13] proposed a maximum likelihood approach to estimate the parameters of a censored bivariate normal distribution. Following their idea, we divide the data into four parts corresponding to the four possible types of pairs of observations:

1. Both observations are above C, $\left(x_{i}^{*}>C_{x},y_{i}^{*}>C_{y}\right)$. There are $n_{11}$ pairs of observations of this type.
2. The first observation is censored and the other one is not, $\left(x_{i}^{*}\leq C_{x},y_{i}^{*}>C_{y}\right)$. There are $n_{01}$ pairs of observations of this type.
3. The first observation is not censored and the other one is censored, $\left(x_{i}^{*}>C_{x},y_{i}^{*}\leq C_{y}\right)$. There are $n_{10}$ pairs of observations of this type.
4. Both observations are censored, $\left(x_{i}^{*}\leq C_{x},y_{i}^{*}\leq C_{y}\right)$. There are $n_{00}$ pairs of observations of this type.

We reorder the data, so that pairs of type 1 come first, followed by types 2, 3, and 4.

$$\left(\begin{array}{ll} x_{1} & y_{1}\\ \vdots & \vdots \\ x_{n_{11}} & y_{n_{11}}\\ x_{n_{11}+1} & y_{n_{11}+1}\\ \vdots & \vdots \\ x_{n_{11}+n_{01}} & y_{n_{11}+n_{01}}\\ x_{n_{11}+n_{01}+1} & y_{n_{11}+n_{01}+1}\\ \vdots & \vdots \\ x_{n_{11}+n_{01}+n_{10}} & y_{n_{11}+n_{01}+n_{10}}\\ x_{n_{11}+n_{01}+n_{10}+1} & y_{n_{11}+n_{01}+n_{10}+1}\\ \vdots & \vdots \\ x_{n_{11}+n_{01}+n_{10}+n_{00}} & y_{n_{11}+n_{01}+n_{10}+n_{00}} \end{array}\right)$$

The maximum likelihood approach of Lyles, Williams, and Chuachoowong [13] uses the equation $f(y_{i},x_{i})=f(y_{i}|x_{i})f(x_{i})$ and the conditional density of a bivariate normal distribution [22]

(2) $$\begin{array}{ll} \left(X^{*}|Y^{*}=y_{i}^{*}\right) & ∼𝒩\left(\mu_{x|y_{i}},\sigma_{x|y}^{2}\right)\\ & ∼𝒩\left(\mu_{x}+\left(\rho \sigma_{x}/\sigma_{y}\right)\left(y_{i}^{*}-\mu_{y}\right),\sigma_{x}^{2}\left(1-\rho^{2}\right)\right) \end{array}$$

This leads to the log‐likelihood function of the reordered data, which is defined as

(3) $$\begin{array}{ll} & \log\;L\left(\theta ;x,y\right)=n_{11}\left(-\log\left(2\pi \sigma_{x}\sigma_{y|x}\right)\right)\\ & \;+\overset{n_{11}}{\underset{i=1}{\sum }}\left(-0.5\left(\frac{{\left(y_{i}-\mu_{y|x_{i}}\right)}^{2}}{\sigma_{y|x}^{2}}+\frac{{\left(x_{i}-\mu_{x}\right)}^{2}}{\sigma_{x}^{2}}\right)\right)\\ & \;+n_{01}\left(-0.5\log(2\pi \sigma_{y}^{2})\right)\\ & \;+\overset{n_{11}+n_{01}}{\underset{i=n_{11}+1}{\sum }}\left(-0.5\frac{{\left(y_{i}-\mu_{y}\right)}^{2}}{\sigma_{y}^{2}}\right)+\left(\overset{n_{11}+n_{01}}{\underset{i=n_{11}+1}{\sum }}\log\Phi \left(\frac{C_{x}-\mu_{x|y_{i}}}{\sigma_{x|y}}\right)\right)\\ & \;+n_{10}\left(-0.5\log\left(2\pi \sigma_{x}^{2}\right)\right)\\ & \;+\overset{n_{11}+n_{01}+n_{10}}{\underset{i=n_{11}+n_{01}+1}{\sum }}\left(-0.5\frac{{\left(x_{i}-\mu_{x}\right)}^{2}}{\sigma_{x}^{2}}\right)+\left(\overset{n_{11}+n_{01}+n_{10}}{\underset{i=n_{11}+n_{01}+1}{\sum }}\log\Phi \left(\frac{C_{y}-\mu_{y|x_{i}}}{\sigma_{y|x}}\right)\right)\\ & \;+n_{00}\left(-0.5\log\left(2\pi \sigma_{y}^{2}\right)\right)\\ & \;+n_{00}\cdot \log\left(\int_{-\infty }^{C_{y}}\exp\left(-\frac{{\left(y-\mu_{y}\right)}^{2}}{2\sigma_{y}^{2}}\right)\cdot \Phi \left(\frac{C_{x}-\left(\mu_{x}+\frac{\rho \sigma_{x}\left(y-\mu_{y}\right)}{\sigma_{y}}\right)}{\sigma_{x}\sqrt{1-\rho^{2}}}\right)dy\right) \end{array}$$

with $\mu_{x|y_{i}}=\mu_{x}+\left(\rho \sigma_{x}/\sigma_{y}\right)\left(y_{i}-\mu_{y}\right)$, $\mu_{y|x_{i}}=\mu_{y}+\left(\rho \sigma_{y}/\sigma_{x}\right)(x_{i}-\mu_{x})$, $\sigma_{x|y}^{2}=\sigma_{x}^{2}\left(1-\rho^{2}\right)$ and $\sigma_{y|x}^{2}=\sigma_{y}^{2}\left(1-\rho^{2}\right)$ [13].

Maximizing the log‐likelihood function leads to an estimate of θ called $\tilde{\theta }$ in each bootstrap sample. This optimization problem can be solved numerically, for example, by using the Nelder‐Mead algorithm from the R package optimx [23], and the integral in the last term of the log‐likelihood function can be approximated by applying the function pmvnorm from the R package mvtnorm [24, 25, 26].

After estimating the parameters of the bivariate normal distribution $\tilde{\theta }$ in each Bootstrap sample, this can be used to estimate the values of the reference lines of the Bland–Altman plot. Following the formulas presented by Bland and Altman [2], the value of the bias line in a sample with $(x_{i},y_{i}),i=1,\ldots ,n$ is calculated as the mean difference $\bar{d}=1/n\cdot \sum_{i=1}^{n}(y_{i}-x_{i})$, the standard deviation of the differences is $s=\sqrt{\sum_{i=1}^{n}{\left(\left(y_{i}-x_{i}\right)-\bar{d}\right)}^{2}/(n-1)}$ and the lower and upper limits of agreement are $a_{l,u}=\bar{d}\pm z_{0.975}s$, assuming normally distributed differences with $z_{0.975}$ equals the 0.975 quantile of the standard normal distribution. Using the estimated parameters of the bivariate normal distribution, the value of the bias line is estimated as the difference between $\tilde{\mu_{y}}$ and $\tilde{\mu_{x}}$: $\tilde{d}=\tilde{\mu_{y}}-\tilde{\mu_{x}}$. An estimator for the standard deviation of the differences is $\tilde{s}=\sqrt{{\tilde{\sigma_{x}}}^{2}+{\tilde{\sigma_{y}}}^{2}-2\tilde{\rho }\tilde{\sigma_{x}}\tilde{\sigma_{y}}}$ and therefore the estimators for the limits of agreement are $\tilde{a_{l,u}}=\tilde{d}\pm z_{0.975}\tilde{s}$. After estimating the parameters of the bivariate normal distribution $\tilde{\theta }$ and the values of the reference lines $\tilde{d}$ and $\tilde{a_{l,u}}$ in each bootstrap sample, we get the final bootstrap estimates $\hat{\theta }$, $\hat{d}$ and $\hat{a_{l,u}}$ by combining the sample estimates [21].

Our next aim is to multiple impute the censored observations j times each with random values taken from the corresponding uncensored bivariate normal distribution using all given information. For the imputation of left‐censored observations of category two $\left(x_{i}^{*}\leq C_{x},y_{i}^{*}>C_{y}\right)$, we apply a similar imputation procedure as described by Lubin et al. [11] using in a two‐step process the cumulative distribution and the quantile function of the distribution of $X_{i}^{*}$ given $Y_{i}^{*}=y_{i\left\{y_{i}^{*}>C_{y}\right\}}^{*}$ which is $𝒩\left(\hat{\mu_{x|y_{i}}},\hat{\sigma_{x|y}^{2}}\right)$ see (2). In the first step, we draw $r_{ij}$ random numbers from a uniform distribution over the interval $\left(\left.0;F_{𝒩\left(\hat{\mu_{x|y_{i}}},\hat{\sigma_{x|y}^{2}}\right)}\left(C_{x}\right)\right]\right.$. In the second step, multiple imputed values of $x_{i}$ are calculated from the quantile function of $𝒩\left(\hat{\mu_{x|y_{i}}},{\hat{\sigma_{x|y}}}^{2}\right)$ at $r_{ij}$. We call the j imputed values of $x_{i}$ $x_{ij}^{mi}$. In a similar manner, it is possible to multiple impute $y_{i}$ to get $y_{ij}^{mi}$. For observations of category four where both observations are left‐censored, $\left(x_{i}^{*}\leq C_{x},y_{i}^{*}\leq C_{y}\right)$, the j imputed values of $x_{i},y_{i}$ called $\left(x_{ij}^{mi},y_{ij}^{mi}\right)$ are drawn randomly from the corresponding right‐truncated bivariate normal distribution with the estimated parameter vector $\hat{\theta }$ and with limits $\left(C_{x},C_{y}\right)$. Here, $\hat{\theta }$ is identical to the above‐described final bootstrap estimate of the bivariate normal distribution.

Now, we have all the elements to draw a Bland–Altman plot for observations with single left‐censoring. The reference lines of the plot are $\hat{d}$ and $\hat{a_{l,u}}$. The uncensored observations are plotted as mean $0.5*\left(x_{i}+y_{i}\right)$ on the x‐axis and as difference $y_{i}-x_{i}$ on the y‐axis. The multiple imputed censored observations are plotted similarly as the mean and as the difference of the imputed values. The Supporting Information provide the relevant R code to compute a Bland–Altman plot for data originating from a censored bivariate lognormal distribution.

### 2.2 Scenario 2: Bland–Altman Plot for Multiple‐Left and Multiple‐Interval Censored Variables

In the second scenario, the censoring limits of the observation pairs are not fixed, but each observation has an individual censoring limit. In addition, interval‐censored observations are available for one of the variables. In mathematical terms, let X and Y denote two random variables. The variable X is subject to multiple left‐censoring, and Y is subject to both multiple left‐censoring and multiple interval‐censoring. We observe a random sample $(x_{i},y_{i}),i=1,\ldots ,n$ with thresholds for left‐censoring $C_{x_{i}}$ and $C_{y_{i1}}$ and with lower and upper thresholds for interval‐censoring $C_{y_{i1}}$ and $C_{y_{i2}}$ for the ith observation. We assume that $X^{*}$ and $Y^{*}$ are the corresponding latent variables of X and Y with

$$x_{i}=\left\{\begin{array}{cc} x_{i}^{*} & \text{if}\;x_{i}^{*}>C_{x_{i}}\\ \text{left‐censored} & \text{if}\;x_{i}^{*}\leq C_{x_{i}} \end{array}\right.$$

and

$$y_{i}=\left\{\begin{array}{cc} y_{i}^{*} & \text{if}\;y_{i}^{*}>C_{y_{i2}}\;\;\;\\ \text{left‐censored} & \text{if}\;y_{i}^{*}\leq C_{y_{i1}}\;\;\;\\ \text{interval‐censored} & \text{if}\;C_{y_{i1}}\leq y_{i}^{*}\leq C_{y_{i2}} \end{array}\right.$$

Similarly to scenario 1, we assume that $\left(X^{*},Y^{*}\right)$ follow a bivariate normal distribution with the parameter vector $\theta ={\left(\mu_{x},\mu_{y},\sigma_{x}^{2},\sigma_{y}^{2},\rho \right)}^{T}$.

The next steps to derive all elements to draw a Bland–Altman plot are very similar to those described in the first scenario 2.1. We start with drawing bootstrap samples. In each sample, the parameters θ of the assumed bivariate normal distribution and the values of the reference lines of the Bland–Altman plot are estimated. For the estimation of θ, we develop and expand the maximum likelihood approach of Lyles, Williams, and Chuachoowong [13] applied in Section 2.1.

In each bootstrap sample, the dataset is partitioned into six distinct segments, each corresponding to one of the six possible categories of observation pairs. Subsequently, the data is reordered according to these categories:

1. Both observations are not censored. There are $n_{11}$ pairs of observations of this type.
2. The first observation is left‐censored and the other one not, $\left(x_{i}^{*}\leq C_{x_{i}},y_{i}^{*}>C_{y_{i2}}\right)$. There are $n_{01}$ pairs of observations of this type.
3. The first observation is not censored and the other one is left‐censored, $\left(x_{i}^{*}>C_{x_{i}},y_{i}^{*}\leq C_{y_{i1}}\right)$. There are $n_{10}$ pairs of observations of this type.
4. Both observations are left‐censored, $\left(x_{i}^{*}\leq C_{x_{i}},y_{i}^{*}\leq C_{y_{i1}}\right)$. There are $n_{00}$ pairs of observations of this type.
5. The first observation is not censored and the other one is interval‐censored, $\left(x_{i}^{*}>C_{x_{i}},C_{y_{i1}}\leq y_{i}^{*}\leq C_{y_{i2}}\right)$. There are $n_{12}$ pairs of observations of this type.
6. The first observation is left‐censored and the other one is interval‐censored, $\left(x_{i}^{*}\leq C_{x_{i}},C_{y_{i1}}\leq y_{i}^{*}\leq C_{y_{i2}}\right)$. There are $n_{02}$ pairs of observations of this type.

The likelihood function is derived in a manner analogous to the first scenario and Lyles, Williams, and Chuachoowong [13]. For observations of type 5, we use the equation $P(C_{y_{1}}\leq Y^{*}\leq C_{y_{2}}|X^{*})=P(Y^{*}\leq C_{y_{2}}|X^{*})-P(C_{y_{1}}\leq Y^{*}|X^{*})$ to derive the likelihood function. The log‐likelihood function of the reordered data for censored variables is

(4) $$\begin{array}{ll} & \log\;L\left(\theta ;x,y\right)=n_{11}\left(-\log\left(2\pi \sigma_{x}\sigma_{y|x}\right)\right)\\ & \;+\overset{n_{11}}{\underset{i=1}{\sum }}\left(-0.5\left(\frac{{\left(y_{i}-\mu_{y|x_{i}}\right)}^{2}}{\sigma_{y|x}^{2}}+\frac{{\left(x_{i}-\mu_{x}\right)}^{2}}{\sigma_{x}^{2}}\right)\right)\\ & \;+n_{01}\left(-0.5\log(2\pi \sigma_{y}^{2})\right)\\ & \;+\overset{n_{11}+n_{01}}{\underset{i=n_{11}+1}{\sum }}\left(-0.5\frac{{\left(y_{i}-\mu_{y}\right)}^{2}}{\sigma_{y}^{2}}\right)+\left(\overset{n_{11}+n_{01}}{\underset{i=n_{11}+1}{\sum }}\log\Phi \left(\frac{C_{x_{i}}-\mu_{x|y_{i}}}{\sigma_{x|y}}\right)\right)\\ & \;+n_{10}\left(-0.5\log\left(2\pi \sigma_{x}^{2}\right)\right)\\ & \;+\overset{n_{11}+n_{01}+n_{10}}{\underset{i=n_{11}+n_{01}+1}{\sum }}\left(-0.5\frac{{\left(x_{i}-\mu_{x}\right)}^{2}}{\sigma_{x}^{2}}\right)+\left(\overset{n_{11}+n_{01}+n_{10}}{\underset{i=n_{11}+n_{01}+1}{\sum }}\log\Phi \left(\frac{C_{y_{i1}}-\mu_{y|x_{i}}}{\sigma_{y|x}}\right)\right)\\ & \;+n_{00}\left(-0.5\log\left(2\pi \sigma_{y}^{2}\right)\right)\\ & \;+\overset{n_{11}+n_{01}+n_{10}+n00}{\underset{i=n_{11}+n_{01}+n_{10}+1}{\sum }}\log\left(\int_{-\infty }^{C_{y_{i1}}}\exp\left(-\frac{{\left(y-\mu_{y}\right)}^{2}}{2\sigma_{y}^{2}}\right)\right.\\ & \;\cdot \left.\Phi \left(\frac{C_{x_{i}}-\left(\mu_{x}+\frac{\rho \sigma_{x}\left(y-\mu_{y}\right)}{\sigma_{y}}\right)}{\sigma_{x}\sqrt{1-\rho^{2}}}\right)dy\right)\\ & \;+n_{12}\left(-0.5\log\left(2\pi \sigma_{x}^{2}\right)\right)\\ & \;+\overset{n_{11}+n_{01}+n_{10}+n_{00}+n_{12}}{\underset{i=n_{11}+n_{01}+n_{10}+n_{00}+1}{\sum }}\left(-0.5\frac{{\left(x_{i}-\mu_{x}\right)}^{2}}{\sigma_{x}^{2}}\right)\\ & \;+\left(\overset{n_{11}+n_{01}+n_{10}+n_{00}+n_{12}}{\underset{i=n_{11}+n_{01}+n_{10}+n_{00}+1}{\sum }}\log\left(\Phi \left(\frac{C_{y_{i2}}-\mu_{y|x_{i}}}{\sigma_{y|x}}\right)\right.\right.\\ & \;\left.\left.-\;\Phi \left(\frac{C_{y_{i1}}-\mu_{y|x_{i}}}{\sigma_{y|x}}\right)\right)\right)\\ & \;+n_{02}\left(-0.5\log\left(2\pi \sigma_{y}^{2}\right)\right)\\ & \;+\overset{n_{11}+n_{01}+n_{10}+n_{00}+n_{12}+n_{02}}{\underset{i=n_{11}+n_{01}+n_{10}+n_{00}+n_{12}+1}{\sum }}\log\left(\int_{C_{y_{i1}}}^{C_{y_{i2}}}\exp\left(-\frac{{\left(y-\mu_{y}\right)}^{2}}{2\sigma_{y}^{2}}\right)\right.\\ & \;\cdot \left.\Phi \left(\frac{C_{x_{i}}-\left(\mu_{x}+\frac{\rho \sigma_{x}\left(y-\mu_{y}\right)}{\sigma_{y}}\right)}{\sigma_{x}\sqrt{1-\rho^{2}}}\right)dy\right) \end{array}$$

with $\mu_{x|y_{i}}=\mu_{x}+\left(\rho \sigma_{x}/\sigma_{y}\right)\left(y_{i}-\mu_{y}\right)$, $\mu_{y|x_{i}}=\mu_{y}+\left(\rho \sigma_{y}/\sigma_{x}\right)(x_{i}-\mu_{x})$, $\sigma_{x|y}^{2}=\sigma_{x}^{2}\left(1-\rho^{2}\right)$ and $\sigma_{y|x}^{2}=\sigma_{y}^{2}\left(1-\rho^{2}\right)$.

Maximizing this log‐likelihood function by numerical optimization yields an estimate for θ called $\tilde{\theta }$. Analogous to the approach in scenario 1 (2.1) the value of the bias line is estimated as $\tilde{d}=\tilde{\mu_{y}}-\tilde{\mu_{x}}$ and the limits of agreement as $\tilde{a_{l,u}}=\tilde{d}\pm z_{0.975}\tilde{s}$ in each bootstrap sample. After that, these estimates are combined to get final bootstrap estimates $\hat{\theta }$, $\hat{d}$, and $\hat{a_{l,u}}$. Then the censored observations are multiple imputed according to the estimated distribution and all other given information.

The multiple imputation of left‐censored observations of category two $\left(x_{i}^{*}\leq C_{x_{i}},y_{i}^{*}>C_{y_{i2}}\right)$, three $\left(x_{i}^{*}>C_{x_{i}},y_{i}^{*}\leq C_{y_{i1}}\right)$, and four $\left(x_{i}^{*}\leq C_{x_{i}},y_{i}^{*}\leq C_{y_{i1}}\right)$ is identical to the first scenario (2.1). Interval censored observations of category five $\left(x_{i}^{*}>C_{x_{i}},C_{y_{i1}}\leq y_{i}^{*}\leq C_{y_{i2}}\right)$ are multiple imputed through a two‐step process. In the first step random numbers $r_{ij}$ are drawn from a uniform distribution with limits at the values of $\left(C_{y_{1}},C_{y_{2}}\right)$ of the distribution function of $𝒩\left(\hat{\mu_{y|x_{i}}},\hat{\sigma_{y|x}^{2}}\right)$:

(5) $$R_{ij}∼𝒰\left(F_{𝒩\left(\hat{\mu_{y|x_{i}}},\hat{\sigma_{y|x}^{2}}\right)}\left(C_{y_{i1}}\right);F_{𝒩\left(\hat{\mu_{y|x_{i}}},\hat{\sigma_{y|x}^{2}}\right)}\left(C_{y_{i2}}\right)\right)$$

Then the multiple imputed values of $y_{ij}^{mi}$ are calculated from the quantile function of $𝒩\left(\hat{\mu_{y|x_{i}}},\hat{\sigma_{y|x}^{2}}\right)$ at values $r_{ij}$

(6) $$y_{ij}^{mi}=F_{𝒩\left(\hat{\mu_{y|x_{i}}},\hat{\sigma_{y|x}^{2}}\right)}^{-1}\left(r_{ij}\right)$$

Censored observations from category six $\left(x_{i}^{*}\leq C_{x_{i}},C_{y_{i1}}\leq y_{i}^{*}\leq C_{y_{i2}}\right)$ are drawn at random from a truncated bivariate normal distribution with parameter $\hat{\theta }$ and lower limits $\left(-\infty ;C_{y_{i1}}.\right]$ and upper limits $\left(C_{x_{i}};C_{y_{i2}}.\right]$. With these elements, the Bland–Altman plot can be drawn. The Supporting Information include the corresponding R code for constructing Bland–Altman plots for observations from a censored bivariate lognormal distribution.

## 3 Simulation Study

### 3.1 A Comparison of Bland–Altman Plots for Single‐Left Censored Variables Using a Simulated Dataset

**FIGURE 1:**
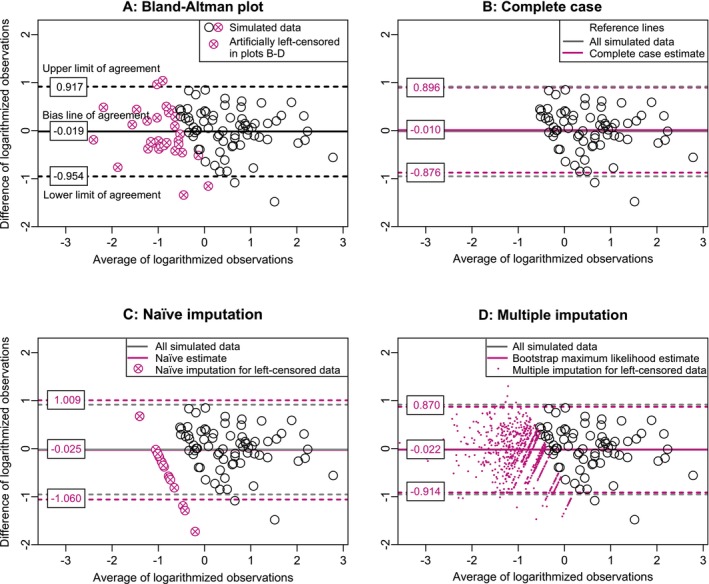
Bland–Altman plots of a bivariate lognormally distributed data set $N=100,\mu_{x}=\mu_{y}=0,\sigma_{x}=\sigma_{y}=1,\rho =0.9$. (A) Shows the classical Bland–Altman plot according to Bland and Altman with the complete data. In (B–D) x=10% and y=30%, of the observations are left‐censored and different approaches are presented: (B) complete case analysis, (C) naïve imputation with half of the censoring cut‐off, and (D) multiple imputation approach.

To illustrate the different approaches to draw a Bland–Altman plot with censored variables, Figure 1 shows four different variants of the Bland–Altman plot. First, the Bland–Altman plot is shown for a simulated bivariate lognormally distributed data set of sample size N=100 with parameters $\mu_{x}=\mu_{y}=0,\sigma_{x}=\sigma_{y}=1,$ and ρ=0.9 without any censoring. The reference lines plotted were estimated according to the specifications from the original work of Bland and Altman. No data were censored for this plot, but for the following graphs, it is assumed that 10% of the observations of x and 30% of those of y are censored. The observations censored in the other plots are already color‐coded in Figure 1A.

Figure 1B shows the results of a complete case analysis. In the Bland–Altman plot, compared to Figure 1A, one can see that all censored observations are missing from the figure and therefore an incomplete picture of the data is shown. In this example, the bias line is somewhat higher and the limits of agreement are narrower, if we compare the reference lines of the Bland–Altman plot of the complete data (grey) with the estimators of the complete case analysis (colored).

In the Bland–Altman plot based on the naïve imputation approach (Figure 1C), all observations are shown, but the censored observations appear lined up due to simple replacement with half the censoring limit. The variability of these observations is not shown. In this example, the limits of agreement are wider with naïve imputation than with the Bland–Altman plot of the complete data. The estimation of the bias line is very similar to the complete data.

Figure 1D shows the Bland–Altman plot after estimation of the reference lines and multiple imputation of the censored observations with the methods described in Section 2.1. Each censored observation (x,y) is represented by 25 imputed values (small colored dots). If only one of the observations x or y was censored, the imputed values lie on a line whose course is determined by the measured value of the uncensored observation. In this figure, all observations are shown along with their variability. The estimation of the bias line is very similar to the bias line of the complete data. The limits of agreement are somewhat narrower. Replication codes for Figure 1D are available in R in the Supporting Information.

### 3.2 Simulation‐Based Evaluation of Reference Line Estimation in Bland–Altman Plots for Single‐Left Censored Variables

Besides this example on a single simulated dataset for demonstration purposes, the result of an extensive simulation study on the estimation of the reference lines is also important to compare the different approaches to draw a Bland–Altman plot with censored observations. We compared the performance of three different estimation methods for the reference lines of the Bland–Altman plot: Complete case analysis, naïve imputation with half the censoring limit, and our multiple imputation approach based on maximum likelihood estimation (see Section 2.1). For the simulation, we used bivariate lognormally distributed data with parameters $\mu_{x}=\mu_{y}=0,\sigma_{x}=\sigma_{y}=1,\rho =0.9$. For three different sample sizes (N=30,50,100), we considered five different censoring scenarios. In the first scenario, 10% of the observations of variable X and 10% of the observations of variable Y were left‐censored. In the next three scenarios, the proportion of left‐censoring of variable Y increased to 20%, 30%, or 40%. In the fifth scenario, 30% of the observations of variable X and 30% of the observations of variable Y were left‐censored. The reference lines were estimated with all three methods and compared to the theoretical value derived from the bivariate lognormal distribution used. To evaluate the performance of the different methods, bias and mean square error were determined based on the results of 2000 iterations for each of the 15 scenarios.

**FIGURE 2:**
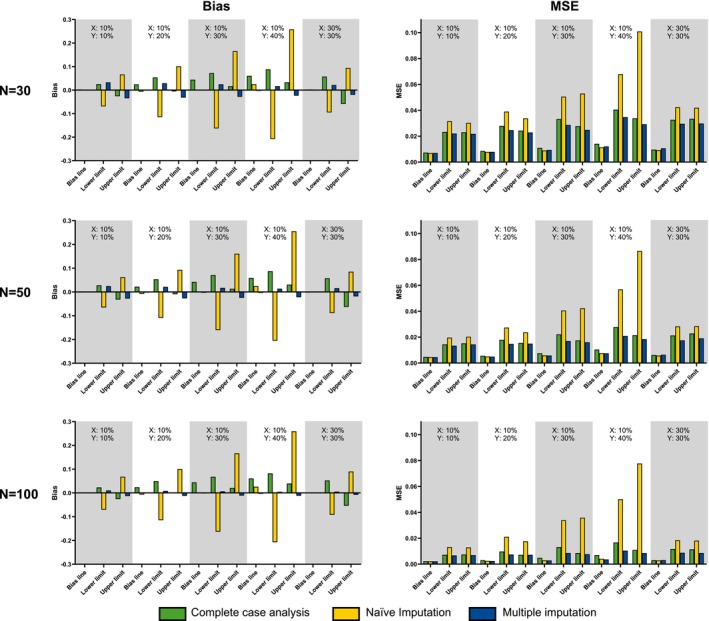
Bias and mean square error (MSE) of three different estimation methods (complete case analysis, naïve imputation with half of the censoring cut‐off, multiple imputation approach) for the reference lines of the Bland–Altman plot from a simulation study with bivariate lognormally distributed data ($\mu_{x}=\mu_{y}=0,\sigma_{x}=\sigma_{y}=1,\rho =0.9$) with 2000 iterations for each scenario. The 15 scenarios differed by samples size (N=30,50, or 100) and the proportion of left‐censored observations per variable x=10% or 30% and y=10%,20%,30%, or 40%.

The simulation results in Figure 2 show the estimated bias and mean square error (MSE) of the different estimation methods. Looking at different sample sizes, the results in the simulated scenarios are quite similar. An increase in the sample size leads to lower MSE values and, therefore, to higher accuracy and precision of the estimates of the reference lines for all estimation methods. An increase in the level of censoring leads to an increase in the absolute value of the bias and an increase in the MSE values for all approaches. In the scenario with a small sample size (N=30) and only few censored observations (X:10% and Y:10% censored), the complete case analysis shows the least bias for the reference lines, though the proposed maximum likelihood estimation approach has lower MSE values than the complete case analysis. With increasing amounts of censoring and increasing sample sizes, the complete case approach usually overestimates the bias line and has a higher bias and MSE than the proposed maximum likelihood estimation approach. The naïve imputation approach with half the determination limit consistently shows higher absolute values of the bias and higher MSE values than the two other approaches for the estimation of the lower and upper limit of agreement of the Bland–Altman diagram. The estimated interval between the lower and upper limit of agreement is wider and overestimates the true interval in most cases. The estimation of the bias line of the Bland–Altman diagram is similarly good as the estimation of the other methods regarding bias and MSE. In the scenarios with equally high censoring rates in X and Y (X:30% and Y:30% censored), the bias of the estimations of the bias line is very small for all estimation approaches. The proposed maximum likelihood estimation approach shows the least absolute value of the bias and the least MSE values for the estimation of the lower and upper limits of agreement in these scenarios. In conclusion, the simulation study shows that the proposed multiple imputation approach based on maximum likelihood estimation is the most robust method overall. In scenarios with small sample sizes and small amounts of censoring, the complete case analysis shows similar good performance in the estimation of the reference lines of the Bland–Altman diagram. In all other scenarios, the complete case analysis is worse. Estimations based on the naïve imputation approach are less reliable than the estimations of any other method.

## 4 Examples With Real Datasets

### 4.1 Comparison of Temperature Effect on Extracts With Allergen Exposures

As an application example for the Bland–Altman diagram for censored observations, we present an allergy‐related example. In a study by Sander and co‐workers [27], the ideal freezing temperatures for long‐term storage of allergen extracts from electrostatic dust collectors were explored, comparing −20°C and −80°C, to prevent protein degradation and ensure precise allergen quantification. Here, results for allergens from dogs (Can f 1) and cats (Fel d 1) were analyzed. To determine the best storage temperature, 152 samples from a field study in households (n=123) and day‐care centers (n=129) were used. The extracts of each sample were divided and frozen at −20°C or −80°C for an average of 1.5 years. Allergens were then quantified by immunoassay and ELISA. The parallel samples were evaluated with the same assay. Further details on the laboratory method are given elsewhere [27].

**FIGURE 3:**
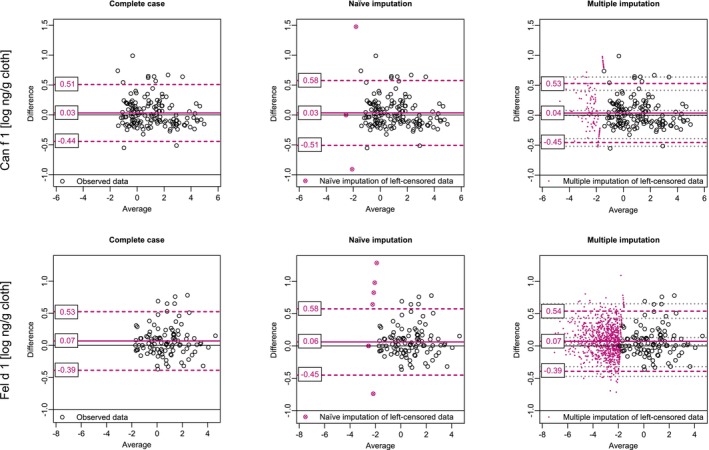
Bland–Altman plots for censored variables of two allergens (Can f 1 and Fel d 1) comparing storage of extracts from electrostatic dust collectors at −20°C and −80°C. Three different approaches are presented: Complete case analysis, naïve imputation with half of the censoring cut‐off, and a multiple imputation approach based on maximum likelihood estimation. For the multiple imputation approach, an underlying bivariate lognormal distribution of the data is assumed for each allergen. Censored observations are multiple imputed according to the estimated distributions and symbolized by 20 imputed values each. Estimated reference lines (and their 95% confidence intervals) are displayed as magenta lines (and grey dashed lines).

The determination limit of the measurement method was fixed and identical for all measurements. For Can f 1 3.3% of observations were left‐censored, and for Fel d 1 28.5%. After examination of the data, a bivariate lognormal distribution was assumed. The comparison of allergen exposures after different storage temperatures using Bland–Altman diagrams is shown in Figure 3. Three different methods to draw a Bland–Altman diagram in case of left‐censored observations are applied: Complete case analysis, naïve imputation with half of the censoring cut‐off, and a multiple imputation approach based on maximum likelihood estimation as described in Section 2.1. The estimators of the bias line are nearly identical across all three methods. The naïve imputation method estimates a much broader interval of agreement compared to the other two methods. Additionally, in the naïve imputation approach, some of the depicted left‐censored observations fall far outside this interval and appear as outliers. For instance, considering one example of Can f 1, the left‐censored observation with a value of −1.80 at the x‐axis (average) and a difference value of 1.48 at the y‐axis (difference) is considerably far from the estimated upper limit of agreement of 0.58. In the multiple imputation approach based on maximum likelihood, the same left‐censored observation is imputed 20 times, and the interval of the depicted imputed values on the y‐axis (difference) ranges from 0.78 to 1.09. Here, the left‐censored observation is also estimated to be above the upper limit of agreement of 0.53, but not as far off as estimated by the naïve imputation approach. In comparison, the Bland–Altman plot based on the complete case analysis does not give any information on the placement of the left‐censored observations. The complete case analysis yields very similar estimates for the reference lines as the maximum likelihood estimates of the multiple imputation method. Unlike the complete case analysis, the maximum likelihood method allows estimation of confidence intervals for the estimators of the reference lines, which is important for the interpretation of the results. For the stored extracts of Can f 1, the Bland–Altman plot did not show a notable difference by temperature. The estimated bias line was close to zero, and the 95% confidence interval for the estimated bias line estimated by maximum likelihood in the multiple imputation approach, included zero. The shape of the point cloud also showed no abnormalities. The stored extracts of Fel d 1 showed a very small difference by temperature: The measurements of the extracts stored at −20°C were on average higher than the measurements of the extracts stored at −80°C with an estimated bias line of 0.07 (0.02–0.13 95% Bootstrap confidence interval estimated by maximum likelihood in the multiple imputation approach). This could suggest a minor increase in the destruction process of proteins due to ice crystal formation and denaturation caused by the storage process at −80°C, as these effects have been reported previously [28].

### 4.2 Comparison of Two Sampler Types to Measure Welding Fumes

An additional example is provided from the WELDOX study, an epidemiological field study in occupational medicine from 2007 to 2009 in Germany [29, 30]. Throughout this study, welding fumes were measured in the inhalable fraction simultaneously using two types of samplers, the GSP 3.5 and the PGP‐EA. Both samplers complied with EN 481 standards [31]. Measurements of the welding fume concentration were conducted in the welders' breathing zone with an average duration of 3.5 h. Filters that reached full saturation were promptly replaced during measurements. The loaded filters were then transported to a laboratory where the welding fumes were quantified by weighing. The welding fume concentration was calculated based on the weight results and the volume of air filtered during the measurement, leading to variable detection limits. Lehnert et al. [29] provides a comprehensive description of the methodology and the study. All participating welders provided written informed consent, and the study received approval from the ethics commission of Ruhr University Bochum.

**FIGURE 4:**
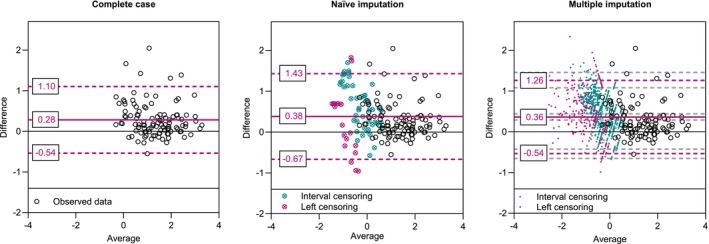
Bland–Altman plots for censored variables of two samplers measuring welding fume. Three different approaches are presented: Complete case analysis, naïve imputation with half of the censoring cut‐off, and a multiple imputation approach based on maximum likelihood estimation. For the multiple imputation approach, an underlying bivariate lognormal distribution of the data is assumed. Censored observations are multiple imputed according to the estimated distribution and symbolized by 15 imputed values each. Estimated reference lines (and their 95% confidence intervals) are displayed as magenta lines (and grey dashed lines).

Of the 176 measurements with each sampler, 15.3% of the observations of the GSP sampler were left‐censored. For the PGP‐EA sampler, 11.4% of the observations were left‐censored and 26.7% were interval‐censored. After examining the data, a censored bivariate log‐normal distribution of the observed measurement pairs was assumed. The data were log‐transformed for the statistical analysis. Three different methods for constructing a Bland–Altman diagram in the presence of multiple left‐censored and multiple interval censored observations are applied: Complete‐case analysis, naïve imputation using half of the censoring cut‐off, and a multiple imputation approach based on maximum likelihood estimation as described in Section 2.2. For the Bland–Altman plot with a multiple imputation approach based on maximum likelihood estimation, 15 000 bootstrap samples were drawn to estimate the reference lines and their confidence intervals. Each censored observation was plotted in this Bland–Altman plot with 15 imputations. Figure 4 shows the comparison of the samplers with the three different Bland–Altman plots. All methods show a positive reference line, indicating that the PGP‐EA sampler measurements are, on average, higher than those of the GSP sampler. The 95% bootstrap confidence interval of the multiple imputation procedure ranges from 0.29 to 0.44. The naïve imputation shows the largest estimated mean difference between the samplers (0.38), and the complete case analysis shows the smallest difference (0.28). The limits of agreement are narrowest for the complete case procedure (−0.54 to 1.10) and widest for naïve imputation (−0.67 to 1.43). For the multiple imputation method, the lower limit of agreement is −0.54 (95 CI −0.65 to −0.43) and the upper limit of agreement is 1.26 (95% CI 1.08 to 1.46). When looking at the individual observation pairs, it is noticeable that the naïve imputation often results in large differences between the samplers for the censored observations, which results in the widest limits of agreement of all methods. In addition, the naïve imputation procedure gives the impression that the variance of the difference of the samplers increases at low measured values. In the multiple imputation procedure, there is no clear difference in the spread of deviations of the samplers with changing measurement values. Changes in the distribution of deviations of the samplers are also not apparent in the complete‐case procedure, though the censored observations are missing in this plot, and thus, especially for low measurement values, no complete picture is available. The observed differences between the two samplers in this field study are mainly due to the placement of the samplers on the welders, as described in Lehnert et al. [29]. The PGP‐EA sampler was typically mounted on the right, and the GSP sampler on the left. Since it can be assumed that most welders were right‐handed, the PGP‐EA sampler mounted on the right was presumably closer to the plume at the welding operation and therefore measured higher concentrations. It should be mentioned that this study was not intended to compare these samplers and that an evaluation of different samplers is usually carried out under laboratory conditions with stationary mounted samplers.

## 5 Discussion

We showed a multiple imputation approach to draw Bland–Altman plots in the case of left‐censored variables. In a simulation study, this method was superior to simple ad‐hoc methods for censored data like complete case analysis and naïve imputation. The simple ad‐hoc methods may lead to biased estimates of the reference line of the Bland–Altman plot, and these estimates had higher mean squared errors than the estimates from the multiple imputation approach. In a simulation study, we were able to show that the multiple imputation method presented is suitable for different sample sizes and different proportions of censored observations.

We chose to represent the censored observations by multiple imputed values following the estimated distribution of the data. These plots are intuitive to understand as they look quite similar to the original Bland–Altman plot [1, 2, 3]. The position of the multiply imputed censored observations corresponds to the probable values under the assumed distribution. This results in a visual representation of the information that can be obtained from the observations. In contrast, the Bland–Altman plots based on a complete case analysis or naïve imputation do not visualize the censored observations at all or only as simple, artificial data points. These graphs, therefore, only provide an incomplete picture of the observations, which may lead to incorrect conclusions.

The maximum likelihood estimation of a bivariate normal distribution from left‐censored observations with a fixed censoring limit used in our first scenario was initially developed by Lyles, Williams, and Chuachoowong [13] to determine the correlation between two censored variables. In contrast, we employ the estimated bivariate normal distribution for the estimation of the reference lines of the Bland–Altman plot and the multiple imputation of the censored observations. In the second scenario, we have further developed this approach to handle left‐ and interval‐censored observations with multiple limits, thereby advancing the maximum likelihood approach of Lyles, Williams, and Chuachoowong [13] which handles left‐ and interval‐censored observations with a fixed limit. Though we present an application of this maximum likelihood estimator for the creation of a Bland–Altman plot, this estimator can also be utilized to estimate the correlation for multiple‐censored observations under the assumption of a bivariate normal distribution of the latent, uncensored variables.

To analyze the estimation of the reference lines of the Bland–Altman plot under various conditions, we conducted an extensive simulation study. Across all situations considered, our method for estimating the reference lines with an maximum likelihood approach proved to be robust, while the naïve substitution method and the complete case analysis usually performed worse, accounting for bias and mean squared error. This result is in line with the findings of Lyles, Williams, and Chuachoowong [13], who compared the different estimators for the parameter ρ of the bivariate normal distribution in a simulation study. Additionally, it has been well‐documented that naïve substitution methods are unsuitable for data evaluation in numerous statistical contexts [5, 6, 7, 8, 11, 12, 14]. As we were able to show, this also applies to the Bland–Altman plot for censored variables, where naïve substitution leads to biased estimation of the reference lines and fails to accurately represent the available information on the location of the censored observations. The simulation study revealed that complete case analysis also leads to biased estimates of the reference lines in many situations. Only in cases of small sample sizes with a low censoring rate are the estimates comparable to those obtained by our method with a maximum likelihood approach. However, complete case analysis does not provide a visual representation of the censored observations in the Bland–Altman plot, and thus, we do not recommend its use in these cases either.

The presented approach to draw a Bland–Altman plot for censored observations can be combined with any other multiple imputation method and various estimation techniques, offering a high degree of flexibility. Though, when selecting a multiple imputation method, it is crucial to ensure that the information given by the censored observations and their censoring limits is incorporated in the imputation model and in the process. Here, we aim to outline some alternative approaches. For instance, multiple imputation could be performed using multiple imputation by chained equations (MICE), where each variable is iteratively imputed using a regression model with a censored dependent variable. Lapidus and co‐authors [32] developed such an approach for two left censored assays, and the multiple imputation presented there can be utilized straightforwardly to create Bland–Altman plots for censored observations. First, the censored observations would be multiply imputed using the MICE method developed by Lapidus and colleagues [32]. In the next step, the reference lines can be calculated in the imputed data sets according to the Bland and Altman formulae [1, 2, 3]. These multiple estimators can then be summarized using Rubin's Rule [19] to obtain the final estimators for the reference lines. To visualise the censored observations in the Bland–Altman plot, a subset of the multiply imputed data (approximately 5–25 imputations) can be used. For each pair of censored observations, the difference and mean are calculated as described by Bland and Altman [1, 2, 3] and plotted visually differentiated from the uncensored observations. Thus, a Bland–Altman plot can be created for censored observations with MICE. The MICE approach allows the incorporation of information from additional variables in the multiple imputation. A good overview of what generally needs to be considered when using MICE can be found elsewhere [33]. As another modification, alternative models for censored dependent variables could be incorporated in the MICE approach. Suitable models for censored variables were described, for instance, by Seok and co‐authors [16] for normally distributed or skewed variables. Other estimation methods, such as the Gibbs sampler [20], have also been described for multiple imputation. By using alternative imputation and estimation methods, it is therefore possible to create a Bland–Altman plot for censored observations where the assumption of a bivariate normal distribution is not justified.

When constructing the Bland–Altman plot for censored variables using the proposed multiple imputation approach, the question arises as to how many imputations are sufficient. While in the past, very small numbers of imputations were considered sufficient for multiple imputation approaches (3–10 imputations) [11, 19], Graham, Olchowski and Gilreath [34] demonstrated that a larger number of imputations (20–100) is advisable. White and colleagues [33] also discuss this question. They employed 100 and 500 imputations in their analyses. In the scenarios presented here, a maximum likelihood approach is combined with bootstrapping to determine estimates of the reference lines. For bootstrapping, it is advisable to generate a large number of bootstrap samples (> 1000 samples) when considering confidence intervals. For further details, refer, for example, to Carpenter and Bithell [35]. Consequently, in our application example comparing sampler types to measure welding fumes, we extracted 15 000 bootstrap samples. Stability assessments of the estimators indicated that the reference line estimators were stable at approximately 50 samples for the bias line and approximately 2750 samples for the limits of agreement, considering two significant digits. The associated confidence intervals were all stable with two significant digits at approximately 6000 samples. We recommend evaluating the stability of the estimators with an increasing number of samples to determine an appropriate number of samples for each study individually. Given the high flexibility of our approach and the numerous possible imputation and estimation methods, we do not find it useful to provide a rule of thumb. The number of samples should be sufficient to obtain stable estimates for the reference lines and their confidence intervals if requested. For the visual representation of the censored observations, we suggest using only a small number of imputations (5–25) and small symbols to prevent the censored data from being overly emphasized compared to the uncensored observations, potentially leading to misinterpretations.

One drawback of the multiple imputation approach for constructing Bland–Altman plots for censored variables is the programming and computational effort required. To facilitate the users' the application of our proposed methods, at least for the scenarios presented in this paper, we provide the R code in the Supporting Information.

In conclusion, the presented method is suitable for constructing Bland–Altman plots for censored variables. It yields superior estimators for the reference lines of the Bland–Altman plot and provides a more accurate representation of censored observations compared to simple methods. The method is highly flexible and can be adapted to a wide range of scenarios. The proposed plots for censored variables closely resemble the well‐known Bland–Altman plots [1, 2, 3], facilitating interpretation for users already familiar with these plots.

## Conflicts of Interest

The authors declare no conflicts of interest.

## Supporting information


**Data S1.** Supporting Information.

## Data Availability

Research data are not shared.
