# Supplementary material for: Bland–Altman Plot for Censored Variables
Source: Stat Med. 2025 Jun 5;44(13-14):e70147. doi: 10.1002/sim.70147 (PMC12141777; doi:10.1002/sim.70147)
Supplement: Supplementary file 1 — Data S1. Supporting Information. [file SIM-44-0-s001.zip › Lotz_Supplementary1.pdf]

```
# Supplementary Material 1 for
# "Bland-Altman plot for censored variables"
# by Anne Lotz, Thomas Behrens, Karl-Heinz Jöckel, and Dirk Taeger
# ==================================================================
# Program to reproduce the Bland-Altman plot for single left-censored
# data as shown in Figure 1D in the manuscript. The method is
# described in section 2.1 (Scenario 1: Bland-Altman plot for
# single-left censored variables).
# All used functions are included in supplemental material 2.

# ==================================================================
# Contents
# ==================================================================
# 1 Load librarys
# 2 Load functions
# 3 Produce simulated data
# 4 Analyze censored data
# 5 Produce figure



# ==================================================================
# ==================================================================
# 1 Load librarys
# ==================================================================
library(MASS)
library(boot)
library(optimx)
library(mvtnorm)
# ==================================================================



# ==================================================================
# ==================================================================
# 2 Load functions
# ==================================================================

# Functions used
YourPath <- file.path("C:", "tmp")
source(file.path(YourPath, "Sup2-Functions-Bland-Altman-plot-for-censored-
variables.R"))


# ==================================================================
# ==================================================================
# 3 Produce simulated data
# ==================================================================

# ==================================================================
# Simulated data (as shown in Figure 1)
# N=100, bivariate log-normal distribution.
mux <- 0
muy <- 0
sigxsq <- 1
sigysq <- 1
rho <- 0.9
KovMatrix <- matrix(c(sigxsq, rho*sqrt(sigxsq*sigysq), rho*sqrt(sigxsq*sigysq),
sigysq),2,2)
# Simulate data set from a bivariate normal distribution
set.seed(4318)
Sim_BNvtlg<-mvrnorm(100,c(mux, muy), KovMatrix, tol = 1e-6, empirical = FALSE, EISPACK
= FALSE)
# Transformation from normal to lognormal
Sim_BNvtlg <- exp(Sim_BNvtlg)
# Calculation of quantiles according to the simulated data set.
# Needed for artificial censoring of observations.
lodx<-quantile(Sim_BNvtlg[,1], probs=0.1) #X:  10% censored, cut-off
lody<-quantile(Sim_BNvtlg[,2], probs=0.3) #Y:  30% censored, cut-off
```

```
Sim_LOD <- c(lodx, lody)
colnames(Sim_BNvtlg)<-c( "x_ori", "y_ori")

# Calculate bias line of agreement and limits of agreement with
# simulated data (complete, not censored)
BiasLine_compl <- mean(log(Sim_BNvtlg[,2]) - log(Sim_BNvtlg[,1]))
LimitsUpp_compl <- BiasLine_compl + 2 * sd(log(Sim_BNvtlg[,2]) - log(Sim_BNvtlg[,1]))
LimitsLow_compl <- BiasLine_compl - 2 * sd(log(Sim_BNvtlg[,2]) - log(Sim_BNvtlg[,1]))


# BNvtlg_notcens: not censored observations (x,y)
BNvtlg_notcens <- Sim_BNvtlg[Sim_BNvtlg[,2]>lody,] # above LODY
BNvtlg_notcens <- BNvtlg_notcens[BNvtlg_notcens[,1]>lodx,] # above LODX
# BNvtlg_cens: censored observations (x,y)
BNvtlg_cens <- Sim_BNvtlg[Sim_BNvtlg[,1] <= lodx | Sim_BNvtlg[,2] <= lody, ]

# x: simulated censored data, with 10% censoring
x<-Sim_BNvtlg[,1]
x[x<=lodx] <- lodx
# y: simulated censored data, with 30% censoring
y<-Sim_BNvtlg[,2]
y[y<=lody] <- lody

# Remove no longer needed objects
remove(mux,muy, sigxsq, sigysq, rho, KovMatrix )



# ===================================================================
# ===================================================================
# 4 Analyze censored data
# ===================================================================
# Estimation of distribution and reference lines
BootResultMCI_BAP<-Calculation_censBAP(x,y, lodx, lody, NumberBootstrap=5000,
SeedBoot=9875)
# Bias line of agreement (censored data, estimate): BootResultMCI_BAP[16]
# Limits of agreement (censored data, estimate): BootResultMCI_BAP[c(22,25)]


# Multiple imputation of censored data points, 25 times each
x_lower <- x
x_lower[x<=lodx] <- NA
x_upper <- x

y_lower <- y
y_lower[y<=lody] <- NA
y_upper <- y

mImpData <- MultipleImp_censBAP(x_lower,x_upper,y_lower,y_upper,
BootResultMCI_BAP[c(1,4,7,10,13)],NImp=25, MImp.seed=20043)

# Calculate data points for the Bland-Altman plot (difference and mean)
# a) not censored observations
yDiff_notcens <- log(BNvtlg_notcens[,2]) - log(BNvtlg_notcens[,1])
xMean_notcens <- 0.5*(log(BNvtlg_notcens[,1]) + log(BNvtlg_notcens[,2]))
# b) censored observations - multiple imputed
mImpData_cens <- mImpData[mImpData[,8] <= lodx | mImpData[,9] <= lody, ]
yDiff_mImp <- log(mImpData_cens[,9]) - log(mImpData_cens[,8])
xMean_mImp<- 0.5*(log(mImpData_cens[,9]) + log(mImpData_cens[,8]))



# ===================================================================
# ===================================================================
# 5 Produce figure
# ===================================================================

plot(yDiff_notcens ~ xMean_notcens, # not censored data points
     xlim = c(-3, 3), ylim = c(-1.5, 1.5),
```

```
     xlab = "Average of logarithmized observations",
     ylab = "Difference of logarithmized observations",
     main = "D: Multiple imputation")
# multiple imputed data points
points(yDiff_mImp ~ xMean_mImp, pch=19, col=rgb(184, 17, 120, max = 255), cex=6/25)
legend("topright", col=c(rgb(156, 156, 156, max = 255), rgb(184, 17, 120, max = 255),
                         rgb(184, 17, 120, max = 255)),
       cex = 0.9, pch=c(NA,NA,19), lty=c(1, 1, 0),lwd=2,
       legend=c("All simulated data", "Bootstrap maximum likelihood estimate",
                "Multiple imputation for left-censored data"))
# Bias line of agreement (complete data):
abline (h=BiasLine_compl, lty=1, col=rgb(178, 178, 178, max = 255), lwd = 2)
# Limits of agreement (complete data):
abline (h=LimitsLow_compl, lty=2, col=rgb(178, 178, 178, max = 255), lwd = 2)
abline (h=LimitsUpp_compl, lty=2, col=rgb(178, 178, 178, max = 255), lwd = 2)
# Bias line of agreement (censored data, estimate):
abline (h=BootResultMCI_BAP[16], lty=1, col=rgb(184, 17, 120, max = 255), lwd = 2)
# Limits of agreement (censored data, estimate):
abline (h=BootResultMCI_BAP[22], lty=2, col=rgb(184, 17, 120, max = 255), lwd = 2)
abline (h=BootResultMCI_BAP[25], lty=2, col=rgb(184, 17, 120, max = 255), lwd = 2)
# Estimates of reference lines
text(x=-3.1, y=BootResultMCI_BAP[16]-0.1,
     paste0(format(round(BootResultMCI_BAP[16],3),nsmall=3)),
     cex=0.9, col=rgb(184, 17, 120, max = 255), pos = 4)
text(x=-3.1, y=BootResultMCI_BAP[22]-0.1,
     paste0(format(round(BootResultMCI_BAP[22],3),nsmall=3)),
     cex=0.9, col=rgb(184, 17, 120, max = 255), pos = 4)
text(x=-3.1, y=BootResultMCI_BAP[25]-0.1,
     paste0(format(round(BootResultMCI_BAP[25],3),nsmall=3)),
     cex=0.9, col=rgb(184, 17, 120, max = 255), pos = 4)

# Additionally include confidence intervals (not in manuscript figure 1)
# Bias line of agreement (censored data, 95% CI):
abline (h=BootResultMCI_BAP[17], lty=3, col=rgb(184, 17, 120, max = 255), lwd = 2)
abline (h=BootResultMCI_BAP[18], lty=3, col=rgb(184, 17, 120, max = 255), lwd = 2)
text(x=-3.1, y=BootResultMCI_BAP[16]-0.25,
     paste0("[",format(round(BootResultMCI_BAP[17],2),nsmall=2), "; ",
format(round(BootResultMCI_BAP[18],2),nsmall=2), "]"),
     cex=0.9, col=rgb(184, 17, 120, max = 255), pos = 4)

# Limits of agreement (censored data, 95% CI):
abline (h=BootResultMCI_BAP[23], lty=3, col=rgb(184, 17, 120, max = 255), lwd = 2)
abline (h=BootResultMCI_BAP[24], lty=3, col=rgb(184, 17, 120, max = 255), lwd = 2)
text(x=-3.1, y=BootResultMCI_BAP[22]-0.25,
     paste0("[",format(round(BootResultMCI_BAP[23],2),nsmall=2), "; ",
format(round(BootResultMCI_BAP[24],2),nsmall=2), "]"),
     cex=0.9, col=rgb(184, 17, 120, max = 255), pos = 4)
abline (h=BootResultMCI_BAP[26], lty=3, col=rgb(184, 17, 120, max = 255), lwd = 2)
abline (h=BootResultMCI_BAP[27], lty=3, col=rgb(184, 17, 120, max = 255), lwd = 2)
text(x=-3.1, y=BootResultMCI_BAP[25]-0.25,
     paste0("[",format(round(BootResultMCI_BAP[26],2),nsmall=2), "; ",
format(round(BootResultMCI_BAP[27],2),nsmall=2), "]"),
     cex=0.9, col=rgb(184, 17, 120, max = 255), pos = 4)
```
