# Supplementary material for: Bland–Altman Plot for Censored Variables
Source: Stat Med. 2025 Jun 5;44(13-14):e70147. doi: 10.1002/sim.70147 (PMC12141777; doi:10.1002/sim.70147)
Supplement: Supplementary file 1 — Data S1. Supporting Information. [file SIM-44-0-s001.zip › Lotz_Supplementary2.pdf]

```
# Supplementary Material 2 for
# "Bland-Altman plot for censored variables"
# by Anne Lotz, Thomas Behrens, Karl-Heinz Jöckel, and Dirk Taeger
# ======================================================================
# Collection of functions for Bland Altman plot for single-left
# censored variables
# ======================================================================
# Contents
# ======================================================================
# 1.1 Main function: Calculation_censBAP
# 1.2 Data preparation function: PreData_censBAP
# 1.3 Likelihood function: Likelihood_censBAP
# 1.4 Estimation function: EstPar_censBAP
# 1.5 Single imputation function: SingleImp_censBAP
# 1.6 Multiple imputation function: MultipleImp_censBAP
# 1.7 References
# ======================================================================
# Librarys used
# library(MASS)
# library(boot)
# library(optimx)
# library(mvtnorm)
# ======================================================================
# ======================================================================



# ======================================================================
# ======================================================================
# 1.1 Main function: Calculation_censBAP
# ======================================================================
# Main function for Bland-Altman plot for censored variables as described in
# "Bland-Altman plot for censored variables"
# by Anne Lotz, Thomas Behrens, Karl-Heinz Jöckel, and Dirk Taeger.
# ======================================================================
# ARGUMENTS
# x:    vector x
# y:    vector y
# lodx: cut-off for variable x (left-censoring)
# lody: cut-off for variable y (left-censoring)
# NumberBootstrap: Number of boostrap samples
# SeedBoot: Seed for random number genertion
# ======================================================================
# OUTPUT
# BootResult_BAP
# BootResultMCI_BAP
# ======================================================================

Calculation_censBAP<- function(x,y, lodx, lody, NumberBootstrap=25, SeedBoot=NULL){
  # Prepare the data
  n <- length(x)
  logData<-cbind(1:n,rep(log(lodx),n), rep(log(lody),n), log(x), log(y))

  # Bootstrap estimation
  set.seed(SeedBoot)
  BootResult_BAP <- boot(logData, EstPar_censBAP, R=NumberBootstrap, ncpus=2)
  Boot_CI_1 <- boot.ci(BootResult_BAP,  type="perc")
  Boot_CI_2 <- boot.ci(BootResult_BAP,  type="perc", index=2)
  Boot_CI_3 <- boot.ci(BootResult_BAP,  type="perc", index=3)
  Boot_CI_4 <- boot.ci(BootResult_BAP,  type="perc", index=4)
  Boot_CI_5 <- boot.ci(BootResult_BAP,  type="perc", index=5)
  Boot_CI_6 <- boot.ci(BootResult_BAP,  type="perc", index=6)
  Boot_CI_7 <- boot.ci(BootResult_BAP,  type="perc", index=7)
  Boot_CI_8 <- boot.ci(BootResult_BAP,  type="perc", index=8)
  Boot_CI_9 <- boot.ci(BootResult_BAP,  type="perc", index=9)

  BootResultMCI_BAP <-  c(.Internal(mean(BootResult_BAP$t[,1])),
Boot_CI_1$perc[4],Boot_CI_1$perc[5],
                        .Internal(mean(BootResult_BAP$t[,2])),
```

```
Boot_CI_2$perc[4],Boot_CI_2$perc[5],
                        .Internal(mean(BootResult_BAP$t[,3])),
Boot_CI_3$perc[4],Boot_CI_3$perc[5],
                        .Internal(mean(BootResult_BAP$t[,4])),
Boot_CI_4$perc[4],Boot_CI_4$perc[5],
                        .Internal(mean(BootResult_BAP$t[,5])),
Boot_CI_5$perc[4],Boot_CI_5$perc[5],
                        .Internal(mean(BootResult_BAP$t[,6])),
Boot_CI_6$perc[4],Boot_CI_6$perc[5],
                        .Internal(mean(BootResult_BAP$t[,7])),
Boot_CI_7$perc[4],Boot_CI_7$perc[5],
                        .Internal(mean(BootResult_BAP$t[,8])),
Boot_CI_8$perc[4],Boot_CI_8$perc[5],
                        .Internal(mean(BootResult_BAP$t[,9])),
Boot_CI_9$perc[4],Boot_CI_9$perc[5])
  names(BootResultMCI_BAP) <- c(
    "mux_BA_Bootmean", "mux_BA_Bootlowlim", "mux_BA_Bootupplim",
    "muy_BA_Bootmean", "muy_BA_Bootlowlim", "muy_BA_Bootupplim",
    "sigxsq_BA_Bootmean", "sigxsq_BA_Bootlowlim", "sigxsq_BA_Bootupplim",
    "sigysq_BA_Bootmean", "sigysq_BA_Bootlowlim", "sigysq_BA_Bootupplim",
    "rho_BA_Bootmean", "rho_BA_Bootlowlim", "rho_BA_Bootupplim",
    "mDiff_BA_Bootmean", "mDiff_BA_Bootlowlim", "mDiff_BA_Bootupplim",
    "sDiff_BA_Bootmean", "sDiff_BA_Bootlowlim", "sDiff_BA_Bootupplim",
    "upplim_BA_Bootmean", "upplim_BA_Bootlowlim", "upplim_BA_Bootupplim",
    "lowlim_BA_Bootmean", "lowlim_BA_Bootlowlim", "lowlim_BA_Bootupplim")
  return(BootResultMCI_BAP)
  }
# ====================================================================



# ====================================================================
# ====================================================================
# 1.2 Data preparation function: PreData_censBAP
# ====================================================================
# Preparation of data for estimation.
# 1. Formatting  of data
# 2. Generating of starting points for the ML estimation function
# ====================================================================
# ARGUMENTS
# x:    vector x (log)
# y:    vector y (log)
# lodx: cut-off for variable x (left-censoring) (log)
# lody: cut-off for variable y (left-censoring) (log)
# ====================================================================
# OUTPUT (global)
# llodx, llody, n11, n01, n00, n10, n1, n, yxGTlody, yxLElody
# type1, type2, type3, type4, yobs, ynotobs, xobs, xnotobs
# xtype1, xtype2, ytype1, ytype3, InitialValue
#
# The output includes the ordered and categorized observations by censoring.
# For additional information see Section 2.1 of manuscript.
# Additionally "InitialValue" is a vector containing starting values for the
# estimation of the parameters of the bivariate lognormal distribution, which
# will be used in ML estimation function.
# InitialValue:  vector of length 5,
#              (muxest,muyest,sqrt_sigmaxsqest,sqrt_sigmaysqest,tanh_rho)
# ====================================================================
PreData_censBAP <- function (x,y, lodx, lody){
  llodx <<- lodx
  llody <<- lody

  yx <<- cbind(y,x)

  n11<<-sum(yx[,1]>llody & yx[,2]>llodx)
  n01<<-sum(yx[,1]>llody & yx[,2]<=llodx)
  n00<<-sum(yx[,1]<=llody & yx[,2]<=llodx)
  n10<<-sum(yx[,1]<=llody & yx[,2]>llodx)
  n1<<-n11 + n10
```

```
ny_measurable <<- n11+n01
ny_notmeasurable <<- n00+n10
nx_measurable <<- n11+n10
nx_notmeasurable <<- n00+n01
n<<-length(yx[,1])

#yxGTlody: greater than censoring cut-off of y (llody)
if (ny_measurable > 1) {
  yxGTlody <<- yx[yx[,1]>llody,]}
if (ny_measurable == 1) {
  yxGTlody <<- matrix(yx[yx[,1]>llody,],1,2)}
if (ny_measurable == 0) {
  yxGTlody <<- matrix(c(NA,NA),1,2)}
if  (is.vector(yxGTlody)==1) { yxGTlody <- matrix(yxGTlody,1,2)}

#yxLElody: less or equal to censoring cut-off of y (llody)
if (ny_notmeasurable > 1) {
  yxLElody <<- yx[yx[,1]<=llody,]}
if (ny_notmeasurable == 1) {
  yxLElody <<- matrix(yx[yx[,1]<=llody,],1,2)}
if (ny_notmeasurable == 0) {
  yxLElody <<- matrix(c(NA,NA),1,2)}
if (is.vector(yxLElody)==1) { yxLElody <- matrix(yxLElody,1,2)}

# types of pairs of observations according to manuscript section 2.2
if (n11 > 0) {type1<<-yxGTlody[yxGTlody[,2]>llodx,]}
if (n10 > 0) {type2<<-yxLElody[yxLElody[,2]>llodx,]}
if (n01 > 0) {type3<<-yxGTlody[yxGTlody[,2]<=llodx,]}
if (n00 > 0) {type4<<-yxLElody[yxLElody[,2]<=llodx,]}

if (n11 == 1) {type1 <<- matrix(type1,1,2)}
if (n10 == 1) {type2 <<- matrix(type2,1,2)}
if (n01 == 1) {type3 <<- matrix(type3,1,2)}
if (n00 == 1) {type4 <<- matrix(type4,1,2)}

if (n11 == 0) {type1 <<- matrix(c(NA,NA),1,2)}
if (n10 == 0) {type2 <<- matrix(c(NA,NA),1,2)}
if (n01 == 0) {type3 <<- matrix(c(NA,NA),1,2)}
if (n00 == 0) {type4 <<- matrix(c(NA,NA),1,2)}

yobs<<-y[y>llody]
ynotobs<<-y[y<=llody]
xobs<<-x[x>llodx]
xnotobs<<-x[(x<=llodx)]

xtype1<<-type1[,2]
xtype2<<-type2[,2]
ytype1<<-type1[,1]
ytype3<<-type3[,1]


# Calculation of starting points for the estimation
# Use data set with naive single imputation 1/2*LOD
x_converted <- x
x_converted[x_converted<=llodx] <- 0.5*llodx
y_converted <- y
y_converted[y_converted<=llody] <- 0.5*llody
muxest<-.Internal(mean(x_converted))
muyest<-.Internal(mean(y_converted))
sigmaxsqest<-sum((x_converted-muxest*rep(1,n))**2)/n
sigmaysqest<-sum((y_converted-muyest*rep(1,n))**2)/n
numer<-sum((x_converted-muxest*rep(1,n))*(y_converted-muyest*rep(1,n)))
rhoh<-numer/(n*sqrt(sigmaxsqest*sigmaysqest))
tanh_rho<- atanh(rhoh)
sqrt_sigmaxsqest<-sqrt(sigmaxsqest)
sqrt_sigmaysqest<-sqrt(sigmaysqest)

# Starting points for special cases
if (sigmaxsqest == 0) {sigmaxsqest <- 1
```

```
  rhoh = 0.5}
  if (sigmaysqest == 0) {sigmaysqest <- 1
  rhoh = 0.5}
  tanh_rho<- atanh(rhoh)
  if (rhoh > 0.99999) {tanh_rho<- atanh(0.99999)}
  sqrt_sigmaxsqest<-sqrt(sigmaxsqest)
  sqrt_sigmaysqest<-sqrt(sigmaysqest)

  # Output of calculated starting values
  InitialValue<<-c(muxest,muyest,sqrt_sigmaxsqest,sqrt_sigmaysqest,tanh_rho)
}
# =====================================================================



# =====================================================================
# =====================================================================
# 1.3 Likelihood function: Likelihood_censBAP
# =====================================================================
# Likelihood function for estimation of the parameters of a bivariate
# lognormal distribution
#
# The maximum likelihood estimation method of the distribution parameters
# of a censored bivariate normal distribution is based on
# Lyles, R. H.; Williams, J. K.; Chuachoowong, R. (2001):
# Correlating two viral load assays with known detection limits.
# In: Biometrics 57 (4), pp. 1238-1244.
# =====================================================================
# ARGUMENTS
# parms:  parameters of the bivariate lognormal distribution
#
# Observed data has to be saved in a global setting (n11, n01, n00,...), see
# function of data set preparation.
# =====================================================================
# OUTPUT
# Likelihood_output: calculated likelihood with given parameters
# =====================================================================
Likelihood_censBAP <- function(parms){
  muxest<-parms[1]
  muyest<-parms[2]
  sqrt_sigmaxsqest<-parms[3]
  sqrt_sigmaysqest<-parms[4]
  tanh_rho <- parms[5]

  sigmaxsqest<-sqrt_sigmaxsqest*sqrt_sigmaxsqest
  sigmaysqest<-sqrt_sigmaysqest*sqrt_sigmaysqest

  rhoh <- tanh(tanh_rho)
  sigyxsqh <- sigmaysqest * (1-rhoh**2)
  sigxysqh <- sigmaxsqest * (1-rhoh**2)

  # Transform in matrix sigma
  Sigma11 <- sigmaxsqest
  Sigma22 <- sigmaysqest
  Sigma12 <- rhoh * sqrt(sigmaxsqest) * sqrt(sigmaysqest)
  SigmaMatrix <- matrix(c(Sigma11,Sigma12, Sigma12,Sigma22),2,2)
  sigma<-SigmaMatrix

  muyxh_type1 <- muyest*rep(1,n11) + (rhoh*sqrt(sigmaysqest/sigmaxsqest)*(xtype1-
muxest*rep(1,n11)))

  n1 <- n11 + n10
  if (n1 > 0) {
    partial1 <- -.5*n1*log(2*pi*sigmaxsqest)
    partial2 <- (-.5/sigmaxsqest)*sum((xobs-muxest*rep(1,n1))**2)
  } else {
    partial1 <- 0
    partial2 <- 0
  }
```

```
  if (n11 > 0) {
    partial3 <- -.5*n11*log(2*pi*sigmaysqest*(1-rhoh**2))
    partial4a <- -1/(2*sigmaysqest*(1-rhoh**2))
    partial4b <- (rhoh*sqrt(sigmaysqest/sigmaxsqest))*(xtype1-muxest*rep(1,n11))
    partial4c <- ytype1-muyest*rep(1,n11)-partial4b
    partial4 <- partial4a*sum(partial4c**2)
  } else {
    partial3 <- 0
    partial4a <- 0
    partial4b <- 0
    partial4c <- 0
    partial4 <- 0
  }

  if (n10 > 0) {
    partial5a <- (rhoh*sqrt(sigmaysqest/sigmaxsqest))*(xtype2-muxest*rep(1,n10))
    partial5b <- sqrt(sigmaysqest*(1-rhoh**2))

    partial5c <- partial5a/partial5b;
    partial5d <- ((llody-muyest)/partial5b)*rep(1,n10)

    partial5vec <- rep(0, n10)
    for (p in 1:n10){
      partial5vec[p]<-log(pnorm(partial5d[p]-partial5c[p]))
    }

    partial5 <- sum(partial5vec)
  } else {
    partial5a <- 0
    partial5b <- 0
    partial5c <- 0
    partial5d <- 0
    partial5 <- 0
  }

  if (n01 > 0) {
    partial6 <- -.5*n01*log(2*pi*sigmaysqest)
    partial7 <- (-.5/sigmaysqest)*sum((ytype3-muyest*rep(1,n01))**2)

    partial8a <- (rhoh*sqrt(sigmaxsqest/sigmaysqest))*(ytype3-muyest*rep(1,n01))
    partial8b <- sqrt(sigmaxsqest*(1-rhoh**2))

    partial8c <- partial8a/partial8b
    partial8d <- ((llodx-muxest)/partial8b)*rep(1,n01)

    partial8vec <- rep(0, n01)
    for (q in 1:n01){
      partial8vec[q]=log(pnorm(partial8d[q]-partial8c[q]))
    }

    partial8 <- sum(partial8vec)
  } else{
    partial6 <- 0
    partial7 <- 0
    partial8a <- 0
    partial8b <- sqrt(sigmaxsqest*(1-rhoh**2))
    partial8c <- 0
    partial8d <- 0
    partial8 <- 0
  }

  if (n00 > 0) {
    partial4_dummi <- pmvnorm(lower=c(-Inf, -Inf), upper=c(llodx,llody), mean=c(muxest,
muyest), sigma=SigmaMatrix)
    partial4extra<- n00 * log(partial4_dummi[1])
  } else {
    partial4extra <- 0
  }
```

```
  # Likelihood
  Likelihood_output <- -2 * (partial1 + partial2 + partial3 + partial4 + partial5 +
partial6 + partial7 + partial8 + partial4extra)
  return(Likelihood_output)
}
# ====================================================================



# ====================================================================
# ====================================================================
# 1.4 Estimation function: EstPar_censBAP
# ====================================================================
# Maximum likelihood estimation of the parameters of a censored bivariate
# normal distribution for a given data set.
# This function is used within the bootstrap function and gives an estimation
# for a bootstrap data set.
#
# The maximum likelihood estimation method of the distribution parameters
# of a censored bivariate normal distribution is based on
# Lyles, R. H.; Williams, J. K.; Chuachoowong, R. (2001):
# Correlating two viral load assays with known detection limits.
# In: Biometrics 57 (4), pp. 1238-1244.
# ====================================================================
# ARGUMENTS
# DataRec:  Contains the following
#              1. Numbered ID
#              2. LODx: censoring cut-off of x (log)
#              3. LODy: censoring cut-off of y (log)
#              4. x censored -> censored observations are set to the cut-off
#              5. y censored ->  censored observations are set to the cut-off
# Index: Vector of (row)numbers defining data set for analysis
#
# The numbered ID and the index are used to define the bootstrap data set.
# ====================================================================
# OUTPUT
# Estimation_complete: vector of the estimated parameters of the distribution
#                      and the estimates of the reference lines of the
#                      Bland-Altman plot
# ====================================================================

EstPar_censBAP <- function(DataRec, Index){
  # 1. Data set
  Data_chosen <- DataRec[Index,]
  # 2. Prepare the data
  # Global output of (n10, n01, n00, ..., xtype1, ....) and of "InitialValue"
  PreData_censBAP(x=Data_chosen[,4],y=Data_chosen[,5], lodx=Data_chosen[1,2],
lody=Data_chosen[1,3])
  # 3. Estimation of the parameters of the bivariate distribution
  Estimation_Opt<-optimx(par=InitialValue, fn=Likelihood_censBAP, method="Nelder-Mead")
  Estimation_BivDist <- cbind(Estimation_Opt$p1, Estimation_Opt$p2,
Estimation_Opt$p3^2, Estimation_Opt$p4^2, tanh(Estimation_Opt$p5))

  # 4. Estimation of parameters of the reference line of the Bland-Altman plot
  # (bias line and limits of agreement)

  mDiff_BA <- Estimation_BivDist[2] - Estimation_BivDist[1] # bias line
  sDiff_BA <- sqrt(Estimation_BivDist[3] + Estimation_BivDist[4] -
2*Estimation_BivDist[5]*sqrt(Estimation_BivDist[3]*Estimation_BivDist[4]))
  RefLineConst <- qnorm(0.975)
  upplim_BA <- mDiff_BA + RefLineConst*sDiff_BA # upper limit of agreement
  lowlim_BA <- mDiff_BA - RefLineConst*sDiff_BA # lower limit of agreement

  # Remove no longer needed objects
  remove(type1, type2, type3, type4, n00, n01, n1, n10, n11,
         nx_measurable, nx_notmeasurable, ny_measurable, ny_notmeasurable,
         InitialValue, yx, yxGTlody, yxLElody,
         xnotobs, xobs, xtype1, xtype2, ynotobs, yobs, ytype1, ytype3,
         llodx, llody,
```

```
        envir = .GlobalEnv)

  # OUTPUT
  Estimation_complete <- c(Estimation_BivDist, mDiff_BA, sDiff_BA, upplim_BA,
lowlim_BA)
  return(Estimation_complete)
}


# ===================================================================
# ===================================================================
# 1.5 Single imputation function: SingleImp_censBAP
# ===================================================================
# Function to impute the censored observations from a given bivariate
# lognormal distribution function.
# See manuscript section 2.1.
# =========================================================================
# ARGUMENTS
# ParameterEstimate: vector of length five containing
#                       1. Mux
#                       2. Muy
#                       3. Sigma_x squared
#                       4. Sigma_y squared
#                       5. Rho
# DataRecInt:   matrix with columns
#              1. Numbered ID
#              2. log(x_lower) (NA if left-censored)
#              3. log(x_upper)
#              4. log(y_lower) (NA if left-censored)
#              5. log(y_upper)
#              6. censtype
# Imp.seed: Seed for random number genertion
# =========================================================================
# Info to censtype:
# type 1: both not censored
# type 2: x left-censored, y not censored
# type 3: x not censored, y left-censored
# type 4: x left-censored, y left-censored
# =========================================================================
# OUTPUT
# ImpData: single imputed data set
#              1. Numbered ID
#              2. log(x_lower) (NA if left-censored)
#              3. log(x_upper)
#              4. log(y_lower) (NA if left-censored)
#              5. log(y_upper)
#              6. censtype
#              7. log(x) with imputed values
#              8. log(y) with imputed values
# =========================================================================

SingleImp_censBAP <- function(ParameterEstimate, DataRecInt, Imp.seed=NULL){

  # Given parameters
  muxest <- ParameterEstimate[1]
  muyest <- ParameterEstimate[2]
  sigmaxsqest <- ParameterEstimate[3]
  sigmaysqest <- ParameterEstimate[4]
  rhoh <- ParameterEstimate[5]

  mu.est.mvrnorm <- c(muxest, muyest)
  Sigma.est.mvrnorm <- matrix(c(sigmaxsqest,
                                rhoh*sqrt(sigmaxsqest)*sqrt(sigmaysqest),
                                rhoh*sqrt(sigmaxsqest)*sqrt(sigmaysqest),
                                sigmaysqest),2,2)

  #---------
  # number per type
```

```
  # type 1: both not censored
  n11<-sum(DataRecInt[,6]==1)
  # type 2: x left-censored, y not censored
  n01<-sum(DataRecInt[,6]==2)
  # type 3: x not censored, y left-censored
  n10<-sum(DataRecInt[,6]==3)
  # type 4: x left-censored, y left-censored
  n00<-sum(DataRecInt[,6]==4)

  #---------
  # Random data generation of a uniform distribution 0-1 for types 2 and 3
  set.seed(Imp.seed)
  RandNumb_ZuCase <- runif(n01+n10)
  # for each type:
  RandNumb.type2 <- RandNumb_ZuCase[1:n01]
  RandNumb.type3 <- RandNumb_ZuCase[(n01+1) : (n01+n10)]


  #---------
  # Empty list to store imputed datasets
  list.Impdata = list()

  ##################################
  # Single imputation per type

  #---------
  # type 1: both not censored => no imputation
  DataRecInt.type1 <- DataRecInt[DataRecInt[,6]==1,]
  x.type1 <- DataRecInt.type1[, 3]
  y.type1 <- DataRecInt.type1[, 5]
  ImpData.type1 <- cbind(DataRecInt.type1, x.type1, y.type1)
  list.Impdata["type1"] <- list(ImpData.type1)

  #---------
  # type 2: x left-censored, y not censored
  if (n01 > 0) {
    DataRecInt.type2 <- DataRecInt[DataRecInt[,6]==2,]
    if (n01 == 1) {DataRecInt.type2 <- matrix(DataRecInt.type2,1,6)}
    y.type2 <- DataRecInt.type2[, 4]
    x_upper.type2 <- DataRecInt.type2[, 3]
    muh.type2 <- muxest*rep(1,n01) + (rhoh*(sqrt(sigmaxsqest)/sqrt(sigmaysqest))*
(y.type2-(muyest*rep(1,n01))))
    sigsqh.type2<-(1-rhoh^2)*sigmaxsqest
    # Calculation of the distribution function at llodx (x_upper.type2)
    pnorm_LOD2<-pnorm(x_upper.type2,mean=muh.type2, sd=sqrt(sigsqh.type2)*rep(1,n01))
    # Transform the random number, so that it is between 0 and pnorm_LOD2
    dummi.type2 <- RandNumb.type2*pnorm_LOD2
    # Calculate the imputed values from the quantile function
    Imp.type2.x <- qnorm(p=dummi.type2, mean=muh.type2,
sd=sqrt(sigsqh.type2)*rep(1,n01))
    ImpData.type2 <- cbind(DataRecInt.type2, Imp.type2.x, y.type2)
    list.Impdata["type2"] <- list(ImpData.type2)
    remove(DataRecInt.type2, y.type2, x_upper.type2, muh.type2, sigsqh.type2,
pnorm_LOD2, dummi.type2, Imp.type2.x)
  }

  #---------
  # type 3: x not censored, y left-censored
  if (n10 > 0) {
    DataRecInt.type3 <- DataRecInt[DataRecInt[,6]==3,]
    if (n10 == 1) {DataRecInt.type3 <- matrix(DataRecInt.type3,1,6)}
    y_upper.type3 <- DataRecInt.type3[, 5]
    x.type3 <- DataRecInt.type3[, 3]
    muh.type3 <- muyest*rep(1,n10) + (rhoh*(sqrt(sigmaysqest)/sqrt(sigmaxsqest))*
(x.type3-(muxest*rep(1,n10))))
    sigsqh.type3<-(1-rhoh^2)*sigmaysqest
    # Calculation of the distribution function at llody (y_upper.type3)
    pnorm_LOD3<-pnorm(y_upper.type3,mean=muh.type3, sd=sqrt(sigsqh.type3)*rep(1,n10))
    # Transform the random number, so that it is between 0 and pnorm_LOD3
```

```
    dummi.type3 <- RandNumb.type3*pnorm_LOD3
    # Calculate the imputed values from the quantile function
    Imp.type3.y <- qnorm(p=dummi.type3, mean=muh.type3,
sd=sqrt(sigsqh.type3)*rep(1,n10))
    ImpData.type3 <- cbind(DataRecInt.type3, x.type3, Imp.type3.y)
    list.Impdata["type3"] <- list(ImpData.type3)
    remove(DataRecInt.type3, y_upper.type3, x.type3, muh.type3, sigsqh.type3,
pnorm_LOD3, dummi.type3, Imp.type3.y)
  }

  #---------
  # type 4: x left-censored, y left-censored

  if (n00 > 0) {
    DataRecInt.type4 <- DataRecInt[DataRecInt[,6]==4,]
    if (n00 == 1) {DataRecInt.type4 <- matrix(DataRecInt.type4,1,6)}
    x_upper.type4 <- DataRecInt.type4[, 3]
    y_upper.type4 <- DataRecInt.type4[, 5]
    Imp.type4 <- matrix(rep(NA, 2*n00), n00, 2)
    for (i.type4 in 1:n00){
      dummi.BivNvtlg <- mvrnorm(n=500,mu=mu.est.mvrnorm, Sigma=Sigma.est.mvrnorm)
      dummi.BivNvtlg.type4 <- dummi.BivNvtlg[dummi.BivNvtlg[,1]<=x_upper.type4[i.type4]
& dummi.BivNvtlg[,2]<=y_upper.type4[i.type4], ]
      Imp.type4[i.type4,] <- dummi.BivNvtlg.type4[1,]
      remove(dummi.BivNvtlg, dummi.BivNvtlg.type4)
    }
    # Combining the pairs
    ImpData.type4 <- cbind(DataRecInt.type4, Imp.type4)
    list.Impdata["type4"] <- list(ImpData.type4)
    remove(DataRecInt.type4, x_upper.type4, y_upper.type4, Imp.type4)
  }

  # ---------------------------
  # Output
  ImpData <- do.call(rbind, list.Impdata)
  ImpData<-ImpData[order(ImpData[,1]),]
  colnames(ImpData) <- c("ID", "lx_lower", "lx_upper", "ly_lower", "ly_upper",
"censtype", "lx_imputed", "ly_imputed")
  return(ImpData)

}
# ====================================================================



# ====================================================================
# ====================================================================
# 1.6 Multiple imputation function: MultipleImp_censBAP
# ====================================================================
# Function to multiple impute the censored observations from a given
# bivariate lognormal distribution function.
# See manuscript section 2.1.
# ==========================================================================
# ARGUMENTS
#            1. x_lower
#            2. x_upper
#            3. y_lower
#            4. y_upper
# ParameterEstimate: vector of length five containing
#                        1. Mux
#                        2. Muy
#                        3. Sigma_x squared
#                        4. Sigma_y squared
#                        5. Rho
# NImp: number of imputations
# MImp.seed: Seed for random number generation
# ==========================================================================
# OUTPUT
# MultImpData: multiple imputed data set
```

```
# ===========================================================================

MultipleImp_censBAP <- function (x_lower,x_upper,y_lower,y_upper,
ParameterEstimate,NImp=25, MImp.seed=NULL){

  Ndata<-length(x_lower)

  #-------------
  # preData data with additional column [6] indicating the type
  # type 1: both not censored
  # type 2: x left-censored, y not censored
  # type 3: x not censored, y left-censored
  # type 4: x left-censored, y left-censored
  preData <- cbind(1:Ndata, log(x_lower), log(x_upper), log(y_lower), log(y_upper),
rep(NA,Ndata))

  preData[x_lower == x_upper & y_lower == y_upper,6] <- 1
  preData[is.na(x_lower) & y_lower == y_upper,6] <- 2
  preData[x_lower == x_upper & is.na(y_lower),6] <- 3
  preData[is.na(x_lower) & is.na(y_lower),6] <- 4

  for (i in 1:NImp){
    Imp.seed2 <- MImp.seed + i
    ImpData <- SingleImp_censBAP(ParameterEstimate, preData, Imp.seed=Imp.seed2)
    if (i == 1) {mImpData.log <- cbind(rep(1,Ndata),ImpData)}
    if (i > 1)  {mImpData.log <- rbind(mImpData.log, cbind(rep(i,Ndata),ImpData) )}
    remove(ImpData)
  }
  mImpData <- mImpData.log
  mImpData[,3:6] <- exp(mImpData[,3:6])
  mImpData[,8:9] <- exp(mImpData[,8:9])

  colnames(mImpData.log) <- c("Imp", "ID", "lx_lower", "lx_upper", "ly_lower",
"ly_upper", "censtype", "lx_imputed", "ly_imputed")
  colnames(mImpData) <- c("Imp", "ID", "x_lower", "x_upper", "y_lower", "y_upper",
"censtype", "x_imputed", "y_imputed")
  return(mImpData)
}



# ===================================================================
# ===================================================================
# 1.7 References
# ===================================================================
# The bibliography can be found in the main manuscript "Bland-Altman plot
# for censored variables" by Anne Lotz, Thomas Behrens, Karl-Heinz Jöckel,
# and Dirk Taeger.
# The most important references for programming the R-code are:
#
#
# The maximum likelihood estimation method of the distribution parameters
# of a censored bivariate normal distribution is based on
# Lyles, R. H.; Williams, J. K.; Chuachoowong, R. (2001):
# Correlating two viral load assays with known detection limits.
# In: Biometrics 57 (4), pp. 1238–1244.
#
# R Core Team (2023). _R: A Language and Environment for Statistical
# Computing_. R Foundation for Statistical Computing, Vienna, Austria.
# <https://www.R-project.org/>.
#
# library(boot)
# Angelo Canty and Brian Ripley (2022). boot: Bootstrap R (S-Plus)
# Functions. R package version 1.3-28.1.
# Davison, A. C. & Hinkley, D. V. (1997) Bootstrap Methods and Their
# Applications. Cambridge University Press, Cambridge. ISBN 0-521-57391-2
#
# library(optimx)
# John C., Ravi Varadhan (2011). Unifying Optimization Algorithms to
```

```
# Aid Software System Users: optimx for R. Journal of Statistical Software,
# 43(9), 1-14. doi 10.18637/jss.v043.i09.
# John C. Nash (2014). On Best Practice Optimization Methods in R. Journal
# of Statistical Software, 60(2), 1-14. doi 10.18637/jss.v060.i02.

# library(MASS)
# Venables, W. N. & Ripley, B. D. (2002) Modern Applied Statistics with S.
# Fourth Edition. Springer, New York. ISBN 0-387-95457-0
#
# library(mvtnorm)
# Genz A, Bretz F (2009). Computation of Multivariate Normal and
# t Probabilities, series Lecture Notes in Statistics. Springer-Verlag,
# Heidelberg. ISBN 978-3-642-01688-2.
```
