# Supplementary material for: Bland–Altman Plot for Censored Variables
Source: Stat Med. 2025 Jun 5;44(13-14):e70147. doi: 10.1002/sim.70147 (PMC12141777; doi:10.1002/sim.70147)
Supplement: Supplementary file 1 — Data S1. Supporting Information. [file SIM-44-0-s001.zip › Lotz_Supplementary3.pdf]

```
# Supplementary Material 3 for
# "Bland-Altman plot for censored variables"
# by Anne Lotz, Thomas Behrens, Karl-Heinz Jöckel, and Dirk Taeger
# ====================================================================
# Collection of functions for Bland Altman plot for multiple-left
# and multiple-interval censored variables
# ====================================================================
# Contents
# ====================================================================
# 1.1 Main function: Calculation_2_censBAP
# 1.2 Data preparation function: PreData_2_censBAP
# 1.3 Likelihood function: Likelihood_2_censBAP
# 1.4 Estimation function: EstPar_2_censBAP
# 1.5 Single imputation function: SingleImp_2_censBAP
# 1.6 Multiple imputation function: MultipleImp_2_censBAP
# 1.7 References
# ====================================================================
# Librarys used
# library(MASS)
# library(boot)
# library(optimx)
# library(mvtnorm)
# ====================================================================
# ====================================================================


# ====================================================================
# ====================================================================
# 1.1 Main function: Calculation_2_censBAP
# ====================================================================
# Main function for Bland-Altman plot for multiple-left and
# multiple-intervalcensored variables as described in
# "Bland-Altman plot for censored variables"
# by Anne Lotz, Thomas Behrens, Karl-Heinz Jöckel, and Dirk Taeger
# in section 2.2.
# ====================================================================
# ARGUMENTS
# x_lower (NA if left-censored)
# x_upper
# y_lower (NA if left-censored)
# y_upper
# NumberBootstrap: Number of boostrap samples
# SeedBoot: Seed for random number genertion
# ====================================================================
# OUTPUT
# BootResult_BAP
# BootResultMCI_BAP
# ====================================================================

Calculation_2_censBAP<- function(x_lower,x_upper, y_lower, y_upper, NumberBootstrap=25,
SeedBoot=NULL){
  # Prepare the data
  n <- length(x_lower)
  logData<-cbind(1:n,log(x_lower), log(x_upper),log(y_lower), log(y_upper))

  # Bootstrap estimation
  set.seed(SeedBoot)
  BootResult_BAP <<- boot(logData, EstPar_2_censBAP, R=NumberBootstrap, ncpus=2)
  Boot_CI_1 <<- boot.ci(BootResult_BAP,  type="perc")
  Boot_CI_2 <<- boot.ci(BootResult_BAP,  type="perc", index=2)
  Boot_CI_3 <<- boot.ci(BootResult_BAP,  type="perc", index=3)
  Boot_CI_4 <<- boot.ci(BootResult_BAP,  type="perc", index=4)
  Boot_CI_5 <<- boot.ci(BootResult_BAP,  type="perc", index=5)
  Boot_CI_6 <<- boot.ci(BootResult_BAP,  type="perc", index=6)
  Boot_CI_7 <<- boot.ci(BootResult_BAP,  type="perc", index=7)
  Boot_CI_8 <<- boot.ci(BootResult_BAP,  type="perc", index=8)
  Boot_CI_9 <<- boot.ci(BootResult_BAP,  type="perc", index=9)

  BootResultMCI_BAP <-  c(.Internal(mean(BootResult_BAP$t[,1])),
```

```
Boot_CI_1$perc[4],Boot_CI_1$perc[5],
                          .Internal(mean(BootResult_BAP$t[,2])),
Boot_CI_2$perc[4],Boot_CI_2$perc[5],
                          .Internal(mean(BootResult_BAP$t[,3])),
Boot_CI_3$perc[4],Boot_CI_3$perc[5],
                          .Internal(mean(BootResult_BAP$t[,4])),
Boot_CI_4$perc[4],Boot_CI_4$perc[5],
                          .Internal(mean(BootResult_BAP$t[,5])),
Boot_CI_5$perc[4],Boot_CI_5$perc[5],
                          .Internal(mean(BootResult_BAP$t[,6])),
Boot_CI_6$perc[4],Boot_CI_6$perc[5],
                          .Internal(mean(BootResult_BAP$t[,7])),
Boot_CI_7$perc[4],Boot_CI_7$perc[5],
                          .Internal(mean(BootResult_BAP$t[,8])),
Boot_CI_8$perc[4],Boot_CI_8$perc[5],
                          .Internal(mean(BootResult_BAP$t[,9])),
Boot_CI_9$perc[4],Boot_CI_9$perc[5])
  names(BootResultMCI_BAP) <- c(
    "mux_BA_Bootmean", "mux_BA_Bootlowlim", "mux_BA_Bootupplim",
    "muy_BA_Bootmean", "muy_BA_Bootlowlim", "muy_BA_Bootupplim",
    "sigxsq_BA_Bootmean", "sigxsq_BA_Bootlowlim", "sigxsq_BA_Bootupplim",
    "sigysq_BA_Bootmean", "sigysq_BA_Bootlowlim", "sigysq_BA_Bootupplim",
    "rho_BA_Bootmean", "rho_BA_Bootlowlim", "rho_BA_Bootupplim",
    "mDiff_BA_Bootmean", "mDiff_BA_Bootlowlim", "mDiff_BA_Bootupplim",
    "sDiff_BA_Bootmean", "sDiff_BA_Bootlowlim", "sDiff_BA_Bootupplim",
    "upplim_BA_Bootmean", "upplim_BA_Bootlowlim", "upplim_BA_Bootupplim",
    "lowlim_BA_Bootmean", "lowlim_BA_Bootlowlim", "lowlim_BA_Bootupplim")
  return(BootResultMCI_BAP)
}
# ==================================================================

# ==================================================================
# ==================================================================
# 1.2 Data preparation function: PreData_2_censBAP
# ==================================================================
# Preparation of data for estimation.
# 1. Formatting  of data
# 2. Generating of starting points for the ML estimation function
# New:  (1) For various censoring limits
#       (2) For interval-censored data in Y.
#           Number of obs. with interval-censoring: n12 and n02
# ==================================================================
# ARGUMENTS
# x_lower:    vector - lower value of x_i (log)
# x_upper:    vector - upper value of x_i (log)
# y_lower:    vector - lower value of y_i (log)
# y_upper:    vector - upper value of y_i (log)
# If x_i is not censored, than x_lower[i] = x_upper[i]
# If y_i is not censored, than y_lower[i] = y_upper[i]
# If x_i is left-censored, than x_lower[i] = NA and x_upper[i] = cut-off of x_i
(censoring limit)
# If y_i is left-censored, than y_lower[i] = NA and y_upper[i] = cut-off of y_i
(censoring limit)
# If y_i is interval-censored, than y_lower[i] = "lower limit of the censoring interval
of y_i"
#      and y_upper[i] = "upper limit of the censoring interval of y_i"
# ==================================================================
# OUTPUT (global)
# n, n11, n01, n00, n10, n02, n12
# yx_all, type1, type2, type3, type4, type5, type6
# InitialValue
#
# The output includes the ordered and categorized observations by censoring.
# For additional information see manuscript.
# Additionally "InitialValue" is a vector containing starting values for the
# estimation of the parameters of the bivariate lognormal distribution, which
# will be used in ML estimation function.
# InitialValue:  vector of length 5,
#              (muxest,muyest,sqrt_sigmaxsqest,sqrt_sigmaysqest,tanh_rho)
```

```
# ===================================================================
PreData_2_censBAP <- function (x_lower,x_upper, y_lower, y_upper){
  n<<-length(x_lower)

  #-------------
  # yx_all data with additional column [5] indicating the type
  # type 1: both not censored
  # type 2: x left-censored, y not censored
  # type 3: x not censored, y left-censored
  # type 4: x left-censored, y left-censored
  # type 5: x not censored, y interval-censored
  # type 6: x left-censored, y interval-censored

  yx_all <- cbind(y_lower, y_upper,x_lower, x_upper, rep(NA,n))

  yx_all[x_lower == x_upper & y_lower == y_upper,5] <- 1
  yx_all[is.na(x_lower) & y_lower == y_upper,5] <- 2
  yx_all[x_lower == x_upper & is.na(y_lower),5] <- 3
  yx_all[is.na(x_lower) & is.na(y_lower),5] <- 4
  yx_all[x_lower == x_upper & y_lower < y_upper,5] <- 5
  yx_all[is.na(x_lower) & y_lower < y_upper,5] <- 6

  yx_all <<- yx_all

  # type 1: both not censored
  n11<<-sum(yx_all[,5]==1)
  if (n11 > 0) {type1 <<- yx_all[yx_all[,5]==1,]}
  if (n11 == 0) {type1 <<- matrix(c(NA,NA,NA,NA,1),1,5)}
  if (n11 == 1) {type1 <<- matrix(type1,1,5)}

  # type 2: x left-censored, y not censored
  n01<<-sum(yx_all[,5]==2)
  if (n01 > 0) {type2 <<- yx_all[yx_all[,5]==2,]}
  if (n01 == 0) {type2 <<- matrix(c(NA,NA,NA,NA,2),1,5)}
  if (n01 == 1) {type2 <<- matrix(type2,1,5)}

  # type 3: x not censored, y left-censored
  n10<<-sum(yx_all[,5]==3)
  if (n10 > 0) {type3 <<- yx_all[yx_all[,5]==3,]}
  if (n10 == 0) {type3 <<- matrix(c(NA,NA,NA,NA,3),1,5)}
  if (n10 == 1) {type3 <<- matrix(type3,1,5)}

  # type 4: x left-censored, y left-censored
  n00<<-sum(yx_all[,5]==4)
  if (n00 > 0) {type4 <<- yx_all[yx_all[,5]==4,]}
  if (n00 == 0) {type4 <<- matrix(c(NA,NA,NA,NA,4),1,5)}
  if (n00 == 1) {type4 <<- matrix(type4,1,5)}

  # type 5: x not censored, y interval-censored
  n12<<-sum(yx_all[,5]==5)
  if (n12 > 0) {type5 <<- yx_all[yx_all[,5]==5,]}
  if (n12 == 0) {type5 <<- matrix(c(NA,NA,NA,NA,5),1,5)}
  if (n12 == 1) {type5 <<- matrix(type5,1,5)}

  # type 6: x left-censored, y interval-censored
  n02<<-sum(yx_all[,5]==6)
  if (n02 > 0) {type6 <<- yx_all[yx_all[,5]==6,]}
  if (n02 == 0) {type6 <<- matrix(c(NA,NA,NA,NA,6),1,5)}
  if (n02 == 1) {type6 <<- matrix(type6,1,5)}

  #----------
  # Calculation of starting points for the estimation
  # Use data set with naive single imputation 1/2*LOD
  x_converted <- x_upper
  x_converted[yx_all[,5] %in% c(2,4,6) ] <- 0.5*x_upper[yx_all[,5] %in% c(2,4,6)]
  y_converted <- y_upper
  y_converted[yx_all[,5] %in% c(3,4)] <- 0.5*y_upper[yx_all[,5] %in% c(3,4)]
  y_converted[yx_all[,5] %in% c(5,6)] <- 0.5*(y_lower[yx_all[,5] %in% c(5,6)] +
y_upper[yx_all[,5] %in% c(5,6)])
```

```
  muxest<-.Internal(mean(x_converted))
  muyest<-.Internal(mean(y_converted))
  sigmaxsqest<-sum((x_converted-muxest*rep(1,n))**2)/n
  sigmaysqest<-sum((y_converted-muyest*rep(1,n))**2)/n
  numer<-sum((x_converted-muxest*rep(1,n))*(y_converted-muyest*rep(1,n)))
  rhoh<-numer/(n*sqrt(sigmaxsqest*sigmaysqest))
  tanh_rho<- atanh(rhoh)
  sqrt_sigmaxsqest<-sqrt(sigmaxsqest)
  sqrt_sigmaysqest<-sqrt(sigmaysqest)

  # Starting points for special cases
  if (sigmaxsqest == 0) {sigmaxsqest <- 1
  rhoh = 0.5}
  if (sigmaysqest == 0) {sigmaysqest <- 1
  rhoh = 0.5}
  tanh_rho<- atanh(rhoh)
  if (rhoh > 0.99999) {tanh_rho<- atanh(0.99999)}
  sqrt_sigmaxsqest<-sqrt(sigmaxsqest)
  sqrt_sigmaysqest<-sqrt(sigmaysqest)

  # Output of calculated starting values
  InitialValue<<-c(muxest,muyest,sqrt_sigmaxsqest,sqrt_sigmaysqest,tanh_rho)

}
# ==================================================================



# ==================================================================
# ==================================================================
# 1.3.2 Likelihood function: Likelihood_2_censBAP
# ==================================================================
# Likelihood function for estimation of the parameters of a bivariate
# lognormal distribution
#
# The maximum likelihood estimation method of the distribution parameters
# of a censored bivariate normal distribution is based on
# Lyles, R. H.; Williams, J. K.; Chuachoowong, R. (2001):
# Correlating two viral load assays with known detection limits.
# In: Biometrics 57 (4), pp. 1238-1244.
#
# New:  (1) For various censoring limits
#       (2) For interval-censored data in Y.
#           Number of obs. with interval-censoring: n12 and n02
# ==================================================================
# ARGUMENTS
# parms:  parameters of the bivariate lognormal distribution
#
# Observed data has to be saved in a global setting (n11, n01, n00,...), see
# function of data set preparation.
# Interval-censored data: n12 and n02.
# ==================================================================
# OUTPUT
# Likelihood_output: calculated likelihood with given parameters
# ==================================================================
Likelihood_2_censBAP <- function(parms){
  muxest<-parms[1]
  muyest<-parms[2]
  sqrt_sigmaxsqest<-parms[3]
  sqrt_sigmaysqest<-parms[4]
  tanh_rho <- parms[5]

  sigmaxsqest<-sqrt_sigmaxsqest*sqrt_sigmaxsqest
  sigmaysqest<-sqrt_sigmaysqest*sqrt_sigmaysqest

  rhoh <- tanh(tanh_rho)
  sigyxsqh <- sigmaysqest * (1-rhoh**2)
  sigxysqh <- sigmaxsqest * (1-rhoh**2)
```

```
  # Transform in matrix sigma
  #Sigma11 <- sigmaxsqest
  #Sigma22 <- sigmaysqest
  Sigma12 <- rhoh * sqrt_sigmaxsqest * sqrt_sigmaysqest
  SigmaMatrix <- matrix(c(sigmaxsqest,Sigma12, Sigma12,sigmaysqest),2,2)
  sigma<-SigmaMatrix


  # type 1: both not censored
  if (n11 == 0) {partial_t1 <- 0}
  if (n11  > 0) {
    t1_1_mean <- rep(0, n11)
    for (q in 1:n11){
      t1_1_mean[q] <- muyest + rhoh * sqrt(sigmaysqest)/sqrt(sigmaxsqest)* (type1[q,3]-
muxest)
    }
    t1_1 <- -log(2*pi*sqrt_sigmaxsqest*sqrt(sigyxsqh))
    t1_2_vec <- (-0.5*( (((type1[,2]-t1_1_mean)**2) / sigyxsqh) +  (((type1[,4]-
muxest)**2) / sigmaxsqest) ))
    partial_t1 <- n11*t1_1 + sum(t1_2_vec)}


  # type 2: x left-censored, y not censored
  if (n01 == 0) {partial_t2 <- 0 }
  if (n01  > 0) {
    # t2_1: f(Y=y)
    t2_1_mean <- muyest * rep(1,n01)
    t2_1_sd <- sqrt_sigmaysqest * rep(1,n01)
    t2_1 <- sum(dnorm(x=type2[,2], mean=t2_1_mean, sd=t2_1_sd, log=TRUE))
    # t2_2: f(x<=Lx|Y=y)
    t2_2_vec <- rep(0, n01)
    t2_2_mean <- rep(0, n01)
    t2_2_sd <- sqrt(sigxysqh)
    for (q in 1:n01){
      t2_2_mean[q] <- muxest + rhoh * sqrt(sigmaxsqest)/sqrt(sigmaysqest)* (type2[q,2]-
muyest)
      t2_2_vec[q] <- pnorm(q=type2[q,4], mean=t2_2_mean[q], sd=t2_2_sd,
lower.tail=TRUE, log.p=TRUE)
    }
    partial_t2 <- t2_1 + sum(t2_2_vec)
  }

  # type 3: x not censored, y left-censored
  if (n10 == 0) {partial_t3 <- 0}
  if (n10  > 0) {
    # t3_1: f(X=x)
    t3_1_mean <- muxest * rep(1,n10)
    t3_1_sd <- sqrt_sigmaxsqest * rep(1,n10)
    t3_1 <- sum(dnorm(x=type3[,4], mean=t3_1_mean, sd=t3_1_sd, log=TRUE))
    # t3_2: f(y<=Ly|X=x)
    t3_2_vec <- rep(0, n10)
    t3_2_mean <- rep(0, n10)
    t3_2_sd <- sqrt(sigyxsqh)
    for (q in 1:n10){
      t3_2_mean[q] <- muyest + rhoh * sqrt(sigmaysqest)/sqrt(sigmaxsqest)* (type3[q,3]-
muxest)
      t3_2_vec[q] <- pnorm(q=type3[q,2], mean=t3_2_mean[q], sd=t3_2_sd,
lower.tail=TRUE, log.p=TRUE)
    }
    partial_t3 <- t3_1 + sum(t3_2_vec)
  }

  # type 4: x left-censored, y left-censored
  if (n00 == 0) {partial_t4 <- 0}
  if (n00  > 0) {
    t4_vec <- rep(0, n00)
    for (q in 1:n00){
      t4_vec[q] <- log(pmvnorm(lower=c(-Inf,-Inf), upper=c(type4[q,4],type4[q,2]),
mean=c(muxest, muyest), sigma=SigmaMatrix))
```

```
    }
    partial_t4 <- sum(t4_vec)
  }

  # type 5: x not censored, y interval-censored
  if (n12 == 0) {partial_t5 <- 0}
  if (n12  > 0) {
    # t5_1: f(X=x)
    t5_1_mean <- muxest * rep(1,n12)
    t5_1_sd <- sqrt_sigmaxsqest * rep(1,n12)
    t5_1 <- sum(dnorm(x=type5[,4], mean=t5_1_mean, sd=t5_1_sd, log=TRUE))
    # t5_2: f(Low_Ly1<y<=Upp_Ly2|X=x)
    t5_2_vec <- rep(0, n12)
    t5_2_mean <- rep(0, n12)
    t5_2_sd <- sqrt(sigyxsqh)
    for (q in 1:n12){
      t5_2_mean[q] <- muyest + rhoh * sqrt(sigmaysqest)/sqrt(sigmaxsqest)* (type5[q,3]-
muxest)
      t5_2_vec[q] <- log(pnorm(q=type5[q,2], mean=t5_2_mean[q], sd=t5_2_sd,
lower.tail=TRUE) - pnorm(q=type5[q,1], mean=t5_2_mean[q], sd=t5_2_sd, lower.tail=TRUE))
    }
    partial_t5 <- t5_1 + sum(t5_2_vec)
  }

  # type 6: x left-censored, y interval-censored
  if (n02 == 0) {partial_t6 <- 0}
  if (n02  > 0) {
    t6_vec <- rep(0, n02)
    for (q in 1:n02){
      t6_vec[q] <- log(pmvnorm(lower=c(-Inf,type6[q,1]),
upper=c(type6[q,4],type6[q,2]), mean=c(muxest, muyest), sigma=SigmaMatrix))
    }
    partial_t6 <- sum(t6_vec)}

  # Likelihood
  Likelihood_output <- -2 * (partial_t1 + partial_t2 + partial_t3 + partial_t4 +
partial_t5 + partial_t6)
  return(Likelihood_output)
}
# ====================================================================


# ====================================================================
# ====================================================================
# 1.5 Single imputation function: SingleImp_2_censBAP
# ====================================================================
# Function to impute the censored observations from a given bivariate
# lognormal distribution function.
# See manuscript section 2.3.
# ==========================================================================
# ARGUMENTS
# ParameterEstimate: vector of length five containing
#                       1. Mux
#                       2. Muy
#                       3. Sigma_x squared
#                       4. Sigma_y squared
#                       5. Rho
# DataRecInt:   matrix with columns
#              1. Numbered ID
#              2. log(x_lower) (NA if left-censored)
#              3. log(x_upper)
#              4. log(y_lower)
#              5. log(y_upper)
#              6. censtype
# Imp.seed: Seed for random number genertion
# ==========================================================================
# Info to censtype:
# type 1: both not censored
# type 2: x left-censored, y not censored
```

```
# type 3: x not censored, y left-censored
# type 4: x left-censored, y left-censored
# type 5: x not censored, y interval-censored
# type 6: x left-censored, y interval-censored
# ==========================================================================
# OUTPUT
# ImpData: single imputed data set
#              1. Numbered ID
#              2. log(x_lower) (=NA bei Links-Zensierung)
#              3. log(x_upper)
#              4. log(y_lower)
#              5. log(y_upper)
#              6. censtype
#              7. log(x) with imputed values
#              8. log(y) with imputed values
# ==========================================================================

SingleImp_2_censBAP <- function(ParameterEstimate, DataRecInt, Imp.seed=NULL){

  # Given parameters
  muxest <- ParameterEstimate[1]
  muyest <- ParameterEstimate[2]
  sigmaxsqest <- ParameterEstimate[3]
  sigmaysqest <- ParameterEstimate[4]
  rhoh <- ParameterEstimate[5]

  mu.est.mvrnorm <- c(muxest, muyest)
  Sigma.est.mvrnorm <- matrix(c(sigmaxsqest,
                              rhoh*sqrt(sigmaxsqest)*sqrt(sigmaysqest),
                              rhoh*sqrt(sigmaxsqest)*sqrt(sigmaysqest),
                              sigmaysqest),2,2)

  #---------
  # number per type

  # type 1: both not censored
  n11<-sum(DataRecInt[,6]==1)
  # type 2: x left-censored, y not censored
  n01<-sum(DataRecInt[,6]==2)
  # type 3: x not censored, y left-censored
  n10<-sum(DataRecInt[,6]==3)
  # type 4: x left-censored, y left-censored
  n00<-sum(DataRecInt[,6]==4)
  # type 5: x not censored, y interval-censored
  n12<-sum(DataRecInt[,6]==5)
  # type 6: x left-censored, y interval-censored
  n02<-sum(DataRecInt[,6]==6)

  #---------
  # Random data generation of a uniform distribution 0-1 for types 2,3, and 5.
  set.seed(Imp.seed)
  RandNumb_ZuCase <- runif(n01+n10+n12)
  # for each type:
  RandNumb.type2 <- RandNumb_ZuCase[1:n01]
  RandNumb.type3 <- RandNumb_ZuCase[(n01+1) : (n01+n10)]
  RandNumb.type5 <- RandNumb_ZuCase[(n01+n10+1) : (n01+n10+n12)]

  #---------
  # Empty list to store imputed datasets
  list.Impdata = list()

  ##################################
  # Single imputatiom per type

  #---------
  # type 1: both not censored => no imputation
  DataRecInt.type1 <- DataRecInt[DataRecInt[,6]==1,]
  x.type1 <- DataRecInt.type1[, 3]
  y.type1 <- DataRecInt.type1[, 5]
```

```
  ImpData.type1 <- cbind(DataRecInt.type1, x.type1, y.type1)
  list.Impdata["type1"] <- list(ImpData.type1)

  #---------
  # type 2: x left-censored, y not censored
  if (n01 > 0) {
    DataRecInt.type2 <- DataRecInt[DataRecInt[,6]==2,]
    if (n01 == 1) {DataRecInt.type2 <- matrix(DataRecInt.type2,1,6)}
    y.type2 <- DataRecInt.type2[, 4]
    x_upper.type2 <- DataRecInt.type2[, 3]
    muh.type2 <- muxest*rep(1,n01) + (rhoh*(sqrt(sigmaxsqest)/sqrt(sigmaysqest))*
(y.type2-(muyest*rep(1,n01))))
    sigsqh.type2<-(1-rhoh^2)*sigmaxsqest
    # Calculation of the distribution function at llodx (x_upper.type2)
    pnorm_LOD2<-pnorm(x_upper.type2,mean=muh.type2, sd=sqrt(sigsqh.type2)*rep(1,n01))
    # Transform the random number, so that it is between 0 and pnorm_LOD2
    dummi.type2 <- RandNumb.type2*pnorm_LOD2
    # Calculate the imputed values from the quantile function
    Imp.type2.x <- qnorm(p=dummi.type2, mean=muh.type2,
sd=sqrt(sigsqh.type2)*rep(1,n01))
    ImpData.type2 <- cbind(DataRecInt.type2, Imp.type2.x, y.type2)
    list.Impdata["type2"] <- list(ImpData.type2)
    remove(DataRecInt.type2, y.type2, x_upper.type2, muh.type2, sigsqh.type2,
pnorm_LOD2, dummi.type2, Imp.type2.x)
  }

  #---------
  # type 3: x not censored, y left-censored
  if (n10 > 0) {
    DataRecInt.type3 <- DataRecInt[DataRecInt[,6]==3,]
    if (n10 == 1) {DataRecInt.type3 <- matrix(DataRecInt.type3,1,6)}
    y_upper.type3 <- DataRecInt.type3[, 5]
    x.type3 <- DataRecInt.type3[, 3]
    muh.type3 <- muyest*rep(1,n10) + (rhoh*(sqrt(sigmaysqest)/sqrt(sigmaxsqest))*
(x.type3-(muxest*rep(1,n10))))
    sigsqh.type3<-(1-rhoh^2)*sigmaysqest
    # Calculation of the distribution function at llody (y_upper.type3)
    pnorm_LOD3<-pnorm(y_upper.type3,mean=muh.type3, sd=sqrt(sigsqh.type3)*rep(1,n10))
    # Transform the random number, so that it is between 0 and pnorm_LOD3
    dummi.type3 <- RandNumb.type3*pnorm_LOD3
    # Calculate the imputed values from the quantile function
    Imp.type3.y <- qnorm(p=dummi.type3, mean=muh.type3,
sd=sqrt(sigsqh.type3)*rep(1,n10))
    ImpData.type3 <- cbind(DataRecInt.type3, x.type3, Imp.type3.y)
    list.Impdata["type3"] <- list(ImpData.type3)
    remove(DataRecInt.type3, y_upper.type3, x.type3, muh.type3, sigsqh.type3,
pnorm_LOD3, dummi.type3, Imp.type3.y)
  }

  #---------
  # type 4: x left-censored, y left-censored
  if (n00 > 0) {
    DataRecInt.type4 <- DataRecInt[DataRecInt[,6]==4,]
    if (n00 == 1) {DataRecInt.type4 <- matrix(DataRecInt.type4,1,6)}
    x_upper.type4 <- DataRecInt.type4[, 3]
    y_upper.type4 <- DataRecInt.type4[, 5]
    Imp.type4 <- matrix(rep(NA, 2*n00), n00, 2)
    for (i.type4 in 1:n00){
      dummi.BivNvtlg <- mvrnorm(n=500,mu=mu.est.mvrnorm, Sigma=Sigma.est.mvrnorm)
      dummi.BivNvtlg.type4 <- dummi.BivNvtlg[dummi.BivNvtlg[,1]<=x_upper.type4[i.type4]
& dummi.BivNvtlg[,2]<=y_upper.type4[i.type4], ]
      Imp.type4[i.type4,] <- dummi.BivNvtlg.type4[1,]
      remove(dummi.BivNvtlg, dummi.BivNvtlg.type4)
    }
    # Combining the pairs
    ImpData.type4 <- cbind(DataRecInt.type4, Imp.type4)
    list.Impdata["type4"] <- list(ImpData.type4)
    remove(DataRecInt.type4, x_upper.type4, y_upper.type4, Imp.type4)
  }
```

```
  #---------
  # type 5: x not censored, y interval-censored
  if (n12 > 0) {
    DataRecInt.type5 <- DataRecInt[DataRecInt[,6]==5,]
    if (n12 == 1) {DataRecInt.type5 <- matrix(DataRecInt.type5,1,6)}
    y_lower.type5 <- DataRecInt.type5[, 4]
    y_upper.type5 <- DataRecInt.type5[, 5]
    x.type5 <- DataRecInt.type5[, 3]
    muh.type5 <- muyest*rep(1,n12) + (rhoh*(sqrt(sigmaysqest)/sqrt(sigmaxsqest))*
(x.type5-(muxest*rep(1,n12))))
    sigsqh.type5<-(1-rhoh^2)*sigmaysqest
    # Calculation of the distribution function at llody (y_upper.type5)
    pnorm_y_lower<-pnorm(y_lower.type5,mean=muh.type5,
sd=sqrt(sigsqh.type5)*rep(1,n12))
    pnorm_y_upper<-pnorm(y_upper.type5,mean=muh.type5,
sd=sqrt(sigsqh.type5)*rep(1,n12))
    # Transform the random number, so that it is between pnorm_y_lower and
pnorm_y_upper
    dummi.type5 <- RandNumb.type5*(pnorm_y_upper - pnorm_y_lower)+pnorm_y_lower
    # Calculate the imputed values from the quantile function
    Imp.type5.y <- qnorm(p=dummi.type5, mean=muh.type5,
sd=sqrt(sigsqh.type5)*rep(1,n12))
    ImpData.type5 <- cbind(DataRecInt.type5, x.type5, Imp.type5.y)
    list.Impdata["type5"] <- list(ImpData.type5)
    remove(DataRecInt.type5, y_upper.type5, x.type5, muh.type5, sigsqh.type5,
pnorm_y_lower, pnorm_y_upper, dummi.type5, Imp.type5.y)
  }

  #---------
  # type 6: x left-censored, y interval-censored
  if (n02 > 0) {
    DataRecInt.type6 <- DataRecInt[DataRecInt[,6]==6,]
    if (n02 == 1) {DataRecInt.type6 <- matrix(DataRecInt.type6,1,6)}
    x_upper.type6 <- DataRecInt.type6[, 3]
    y_lower.type6 <- DataRecInt.type6[, 4]
    y_upper.type6 <- DataRecInt.type6[, 5]
    Imp.type6 <- matrix(rep(NA, 2*n02), n02, 2)
    for (i.type6 in 1:n02){
      dummi.BivNvtlg <- mvrnorm(n=1500,mu=mu.est.mvrnorm, Sigma=Sigma.est.mvrnorm)
      dummi.BivNvtlg.type6 <- dummi.BivNvtlg[dummi.BivNvtlg[,1]<=x_upper.type6[i.type6]
&

dummi.BivNvtlg[,2]>=y_lower.type6[i.type6] & dummi.BivNvtlg[,2]
<=y_upper.type6[i.type6], ]
      Imp.type6[i.type6,] <- dummi.BivNvtlg.type6[1,]
      remove(dummi.BivNvtlg, dummi.BivNvtlg.type6)
    }
    # Combining the pairs
    ImpData.type6 <- cbind(DataRecInt.type6, Imp.type6)
    list.Impdata["type6"] <- list(ImpData.type6)
    remove(DataRecInt.type6, x_upper.type6, y_lower.type6, y_upper.type6, Imp.type6)
  }
  # --------------------------
  # Output
  ImpData <- do.call(rbind, list.Impdata)
  ImpData<-ImpData[order(ImpData[,1]),]
  colnames(ImpData) <- c("ID", "lx_lower", "lx_upper", "ly_lower", "ly_upper",
"censtype", "lx_imputed", "ly_imputed")
  return(ImpData)
}
# ===========================================================


# ===========================================================
# ===========================================================
# 1.6 Multiple imputation function: MultipleImp_2_censBAP
# ===========================================================
# Function to multiple impute the censored observations from a given
```

```
# bivariate lognormal distribution function.
# See manuscript section 2.2
# ===========================================================================
# ARGUMENTS
#              1. x_lower
#              2. x_upper
#              3. y_lower
#              4. y_upper
# ParameterEstimate: vector of length five containing
#                       1. Mux
#                       2. Muy
#                       3. Sigma_x squared
#                       4. Sigma_y squared
#                       5. Rho
# NImp: number of imputations
# MImp.seed: Seed for random number generation
# ===========================================================================
# OUTPUT
# MultImpData: multiple imputed data set
# ===========================================================================

MultipleImp_2_censBAP <- function (x_lower,x_upper,y_lower,y_upper,
ParameterEstimate,NImp=25, MImp.seed=NULL){

  Ndata<-length(x_lower)

  #-------------
  # preData data with additional column [6] indicating the type
  # type 1: both not censored
  # type 2: x left-censored, y not censored
  # type 3: x not censored, y left-censored
  # type 4: x left-censored, y left-censored
  # type 5: x not censored, y interval-censored
  # type 6: x left-censored, y interval-censored

  preData <- cbind(1:Ndata, log(x_lower), log(x_upper), log(y_lower), log(y_upper),
rep(NA,Ndata))

  preData[x_lower == x_upper & y_lower == y_upper,6] <- 1
  preData[is.na(x_lower) & y_lower == y_upper,6] <- 2
  preData[x_lower == x_upper & is.na(y_lower),6] <- 3
  preData[is.na(x_lower) & is.na(y_lower),6] <- 4
  preData[x_lower == x_upper & y_lower < y_upper,6] <- 5
  preData[is.na(x_lower) & y_lower < y_upper,6] <- 6
  #---------
  preData<<-preData
  #---------
  for (i in 1:NImp){
    Imp.seed2 <- MImp.seed + i
    ImpData <- SingleImp_2_censBAP(ParameterEstimate, preData, Imp.seed=Imp.seed2)
    if (i == 1) {mImpData.log <- cbind(rep(1,Ndata),ImpData)}
    if (i > 1)  {mImpData.log <- rbind(mImpData.log, cbind(rep(i,Ndata),ImpData) )}
    remove(ImpData)
  }
  mImpData <- mImpData.log
  mImpData[,3:6] <- exp(mImpData[,3:6])
  mImpData[,8:9] <- exp(mImpData[,8:9])

  colnames(mImpData.log) <- c("Imp", "ID", "lx_lower", "lx_upper", "ly_lower",
"ly_upper", "censtype", "lx_imputed", "ly_imputed")
  colnames(mImpData) <- c("Imp", "ID", "x_lower", "x_upper", "y_lower", "y_upper",
"censtype", "x_imputed", "y_imputed")
  return(mImpData)
}


# ===================================================================
# ===================================================================
# 1.7 References
```

```
# ====================================================================
# The bibliography can be found in the main manuscript "Bland-Altman plot
# for censored variables" by Anne Lotz, Thomas Behrens, Karl-Heinz Jöckel,
# and Dirk Taeger.
# The most important references for programming the R-code are:
#
#
# The maximum likelihood estimation method of the distribution parameters
# of a censored bivariate normal distribution is based on
# Lyles, R. H.; Williams, J. K.; Chuachoowong, R. (2001):
# Correlating two viral load assays with known detection limits.
# In: Biometrics 57 (4), pp. 1238–1244.
#
# R Core Team (2023). _R: A Language and Environment for Statistical
# Computing_. R Foundation for Statistical Computing, Vienna, Austria.
# <https://www.R-project.org/>.
#
# library(boot)
# Angelo Canty and Brian Ripley (2022). boot: Bootstrap R (S-Plus)
# Functions. R package version 1.3-28.1.
# Davison, A. C. & Hinkley, D. V. (1997) Bootstrap Methods and Their
# Applications. Cambridge University Press, Cambridge. ISBN 0-521-57391-2
#
# library(optimx)
# John C., Ravi Varadhan (2011). Unifying Optimization Algorithms to
# Aid Software System Users: optimx for R. Journal of Statistical Software,
# 43(9), 1-14. doi 10.18637/jss.v043.i09.
# John C. Nash (2014). On Best Practice Optimization Methods in R. Journal
# of Statistical Software, 60(2), 1-14. doi 10.18637/jss.v060.i02.

# library(MASS)
# Venables, W. N. & Ripley, B. D. (2002) Modern Applied Statistics with S.
# Fourth Edition. Springer, New York. ISBN 0-387-95457-0
#
# library(mvtnorm)
# Genz A, Bretz F (2009). Computation of Multivariate Normal and
# t Probabilities, series Lecture Notes in Statistics. Springer-Verlag,
# Heidelberg. ISBN 978-3-642-01688-2.
```
